# Rapid adaptation of signaling networks in the fungal pathogen *Magnaporthe oryzae*

**DOI:** 10.1186/s12864-019-6113-3

**Published:** 2019-10-22

**Authors:** Stefan Bohnert, Luis Antelo, Christiane Grünewald, Alexander Yemelin, Karsten Andresen, Stefan Jacob

**Affiliations:** 1 Institut für Biotechnologie und Wirkstoff-Forschung gGmbH (IBWF), Erwin-Schrödinger-Str. 56, D-67663 Kaiserslautern, Germany; 2 Johannes Gutenberg-University Mainz, Mikrobiologie und Weinforschung am Institut für Molekulare Physiologie, Johann-Joachim-Becherweg 15, D-55128 Mainz, Germany

**Keywords:** Rapid adaptation, HOG pathway, *Magnaporthe oryzae*, Rewiring, Reestablishment of osmoregulation, Epigenetics, Evolution of signaling networks, Suppressor

## Abstract

**Background:**

One fundamental question in biology is how the evolution of eukaryotic signaling networks has taken place. “Loss of function” (lof) mutants from components of the high osmolarity glycerol (HOG) signaling pathway in the filamentous fungus *Magnaporthe oryzae* are viable, but impaired in osmoregulation.

**Results:**

After long-term cultivation upon high osmolarity, stable individuals with reestablished osmoregulation capacity arise independently from each of the mutants with inactivated HOG pathway. This phenomenon is extremely reproducible and occurs only in osmosensitive mutants related to the HOG pathway – not in other osmosensitive *Magnaporthe* mutants. The major compatible solute produced by these adapted strains to cope with high osmolarity is glycerol, whereas it is arabitol in the wildtype strain. Genome and transcriptome analysis resulted in candidate genes related to glycerol metabolism, perhaps responsible for an epigenetic induced reestablishment of osmoregulation, since these genes do not show structural variations within the coding or promotor sequences.

**Conclusion:**

This is the first report of a stable adaptation in eukaryotes by producing different metabolites and opens a door for the scientific community since the HOG pathway is worked on intensively in many eukaryotic model organisms.

## Background

**Fig. 1:**
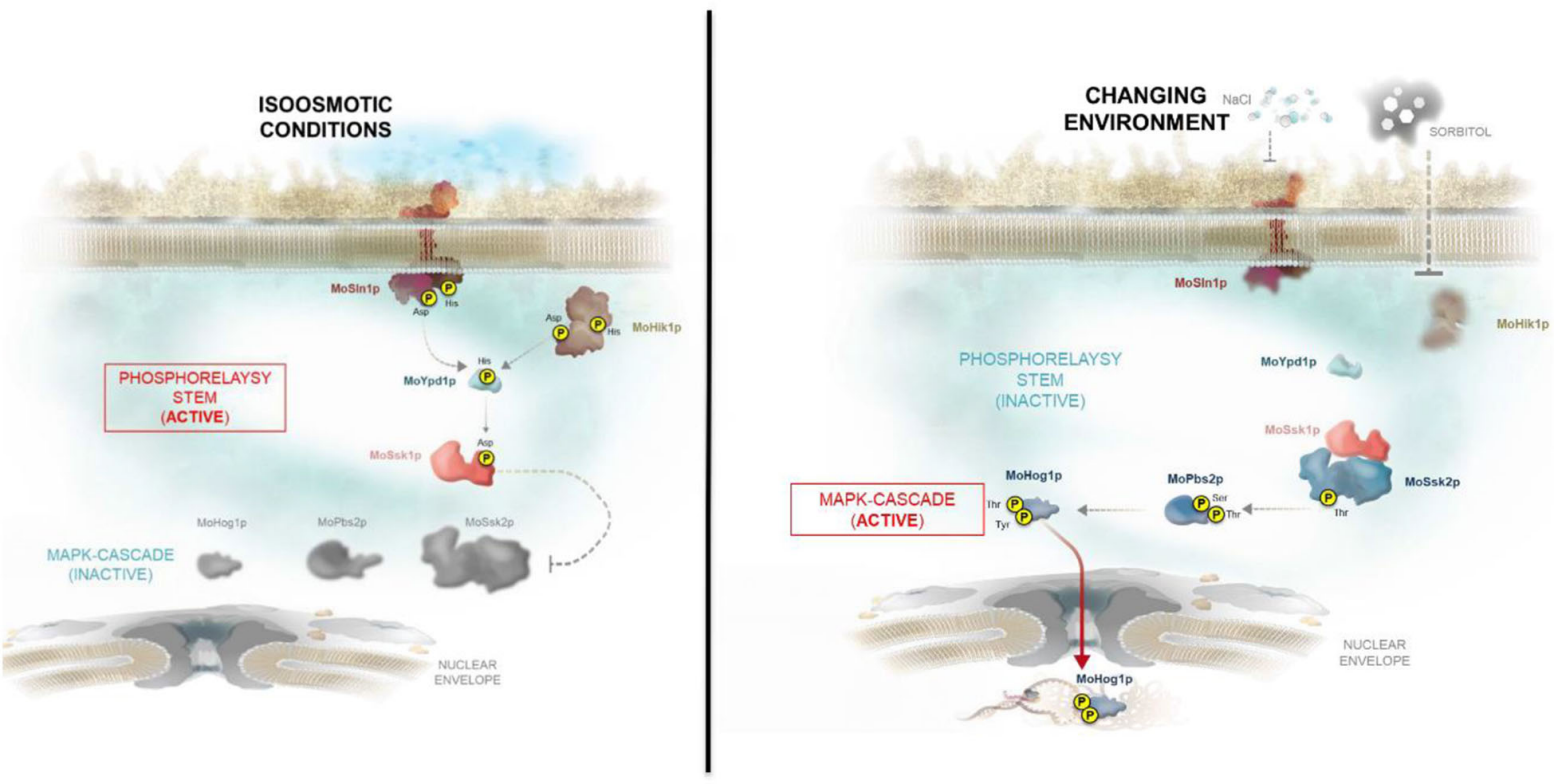
The *Magnaporthe oryzae* HOG signaling cascade. The two-component hybrid histidine kinases MoSln1p and MoHik1p are the sensors for environmental changes. Under normal environmental conditions, the phosphotransferase protein MoYpd1p transfers the phosphate to the response regulator MoSsk1p. Consequently, the phosphorelay system MoSln1p-MoHik1p-MoYpd1p-MoSsk1p is active and the MAPK cascade MoSsk2p-MoPbs2p-MoHog1p is inactive (left). High osmolarity or salt stress leads to dephosphorylation of the sensor kinases MoSln1p and/or MoHik1p resulting in a decrease of MoSsk1p-phosphorylation. Lower phosphorylation-levels at MoSsk2p activate the MAPK cascade MoSsk2p-MoPbs2p-MoHog1p, MoHog1p translocates into the nucleus and activates the cellular reactions

Adaptation is a central biological process that underlies diverse phenomena from the acquisition of antibiotic resistance by microbes to the evolution of niche specialization [1]. Most of these experimental analyses focused on bacterial systems studying the evolution of populations and genetic structures, the adaptive profit of new functions and the consequences of competition dynamics in large populations. The most prominent example to address molecular mechanisms of evolution is the *Escherichia coli* long-term evolution experiment [2]. It reveals complex dynamics, characterized by rapid adaptation, competition between beneficial mutations and extensive genetic parallelism in bacterial populations. However, until now, relatively little has been discovered about the mechanisms of molecular evolutionary adaptation in eukaryotes. One fundamental question in biology is how eukaryotic signaling pathways evolve to adapt towards changing environmental situations. The adaptation of molecular mechanisms from signaling networks and the question why the networks show a high level of complexity are of particular interest [3]. Over the last few years, the understanding of the molecular and biochemical principles of evolution have increased dramatically [4, 5]. Epigenetic modulation, such as DNA methylation, chromatin modification and noncoding RNAs, are found to be involved in the multigenerational transmission of phenotypes and transgenerational inheritance [6]. Nevertheless, there has been little detailed information about how phenotypic changes rest on the rate and the nature of the underlying genotypic or epigenetic changes [7]. Regulatory signaling networks represent the causality of developmental processes. The big challenges regarding regulatory networks generally remain in understanding how genetic variants generate phenotypes [8]. Genomic sequence variants influence the regulation of the expression of genes that encode proteins generating signaling patterns and execute differentiation or even adaptation to changing environments [9]. Microbial signal transduction pathways apply far more modular mechanisms to define their input-output interactions than classical metabolic pathways [10]. Thus, in addition to their core catalytic function, the proteins often contain multiple independently folding domains or motifs that mediate connectivity by interacting with other signaling elements [11]. These elements are found in different combinations with different signaling functions, suggesting insertion and recombination of modules may be a common mechanism of the evolution of new signaling networks [12]. Furthermore, protein domains are extensively recombined in order to facilitate functional innovations [5, 13]. Prominent examples of pathways with a modular architecture mediated by scaffold proteins are signaling pathways in yeast, including several mitogen-activated protein kinase (MAPK) cascades [12, 14]. Attachment of different scaffolds could, in principle, be sufficient to generate new signaling pathways from combinations of preexisting kinases [15]. Canonical MAPK cascades in fungi were reputed to be responsible for a large part of signal transduction. These MAPK pathways contain signal modules in which an activated MAPK kinase kinase (MAPKKK) activates a MAPK kinase (MAPKK), which then activates a MAPK (extracellular signal-regulated kinase: ERK). The MAPK targets comprise transcription factors, phosphatases, protein kinases and other classes of proteins. They regulate physiological processes, cellular morphology, cell cycle progression, metabolism and gene expression in response to various extracellular signals or environmental stresses [16]. Such MAPK pathways appear ideal for exploring the fundamental mechanisms of evolutionary adaptation. Three distinct MAPK genes – *MoKSS1* (formerly *MoPMK1*), *MoSLT2* (formerly *MoMPS1*), and *MoHOG1* (formerly *MoOSM1*) [17] – have been identified in the genome of the filamentous rice blast fungus *Magnaporthe oryzae* (*Pyricularia oryzae*) and possess diverse roles in cellular signaling and pathogenesis-related development [18–20]. The high osmolarity glycerol (HOG) pathway is responsible for osmoregulation in fungi and seems to operate independently of the *MoKSS1* and *MoSLT2* pathways in *M. oryzae* by regulating the biosynthesis of arabitol and glycerol [20]. Upon high external osmolarity, cells have to quickly recover volume by water influx due to the accumulation of compatible solutes, i.e. mainly arabitol in the rice blast fungus. The HOG pathway in *M. oryzae* comprises the MoSln1p/MoHik1p-MoYpd1p-MoSsk1p phosphorelay system and the MAPK cascade MoSsk2p-MoPbs2p-MoHog1 (Fig. 1).

The HOG pathway in *M. oryzae* was previously found to be an attractive system to study the basics of physiological functions of signaling proteins and the mode of action of agricultural fungicides, such as fludioxonil [21–23]. *M. oryzae*-mutants lacking components of the HOG pathway are osmosensitive, since they showed a dramatically reduced ability to accumulate arabitol in the mycelium [20]. In the study presented, osmosensitive “loss of function” (lof) mutants *ΔMohog1*, *ΔMopbs2*, *ΔMossk2*, *ΔMossk1* and *ΔMoypd1* were cultivated upon osmotic stress, resulting in stable individuals outgrowing from the mycelium of each lof mutant after four weeks of stress. These adapted individuals show a reestablished osmoregulation and produce glycerol and not arabitol as the major compatible solute upon osmotic shock. To the best of our knowledge, we present such an independent and stable adaptation of individuals outgrowing from lof mutants for the first time. Furthermore, we believe that this study will open the door for many research groups, since this rapid adaptation phenomenon will be easily addressable in a broad spectrum of eukaryotic model organisms.

## Results

### “Adapted” mutants arise out of independent “loss of function” strains upon long-term cultivation on stress

**Fig. 2:**
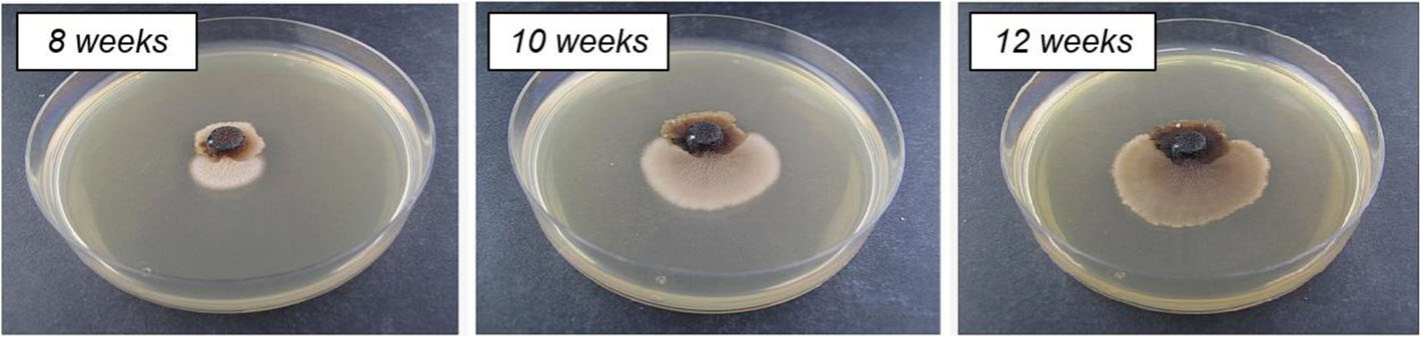
Example of *Magnaporthe oryzae* “loss of function” (lof) strains *ΔMohog1* and *ΔMohog1(adapted)* after long-term cultivation upon salt stress. The lof strain *ΔMohog1* was grown on complete medium (CM) inclusive 1 M KCl at 26 °C. The *ΔMohog1* mutant (dark brownish colony) was found to be highly sensitive towards 1 M KCl, whereas the outgrowing *ΔMohog1(adapted)* strain (bright colony) was able to grow much faster

Apart from molecular biology techniques and next-generation sequencing approaches, classical vegetative growth assays were used to characterize potential functions of genes associated with osmoregulation to study the HOG pathway in *M. oryzae*. Therefore, we cultivated the previously generated lof mutants *ΔMohog1*, *ΔMopbs2*, *ΔMossk2*, *ΔMossk1*, *ΔMoypd1*, *ΔMohik1* and *ΔMosln1* [21] on different stress-inducing media to compare them with the wildtype strain. Mutants with an inactivated HOG pathway are sensitive to osmotic stress and resistant to the fungicide fludioxonil [21]. We noticed in the course of these assays that individual mycelium parts grew out of the sensitive lof mutants *ΔMohog1*, *ΔMopbs2*, *ΔMossk2*, *ΔMossk1* and *ΔMoypd1* after cultivation for at least four to six weeks under continuous salt stress (Fig. 2).

We isolated these individual mycelium parts in order to separate them as pure cultures ready for further investigations and named them *ΔMohog1(adapted)*, *ΔMopbs2(adapted)*, *ΔMossk2(adapted)*, *ΔMossk1(adapted)* and *ΔMoypd1(adapted)*. We have not been able to get adapted strains from the mutants *ΔMosln1* and *ΔMohik1* in the conditions tested so far. That underpins the hypothesis from our previous studies that the two-component hybrid histidine kinase (HK) MoHik1p and the HK MoSln1p can partially take over the function from each other [24] and, thus, the selection pressure maybe not sufficient for an adaptation event in *ΔMosln1* and *ΔMohik1*. Furthermore, it was not possible for us to isolate adapted strains from HOG pathway-independent osmosensitive *Magnaporthe*-mutant strains, i.e. *ΔMostu1* (transcription factor in cAMP/PKA signaling pathway, *ΔMogpd1* (glycerol-3-phosphate dehydrogenase) or *ΔMoskn7* (response regulator protein). Of course, we checked in the adapted strains whether the genes originally inactivated in the “parent” lof mutants were still inactivated in order to avoid any possibility of contaminations or confusions about mixed cultures. We did this for all adapted strains using ITS-sequencing and southern blot analyses (Additional file 2: Figure S1).

In empirical investigations, a combination of different vegetative growth assays was used to characterize the “rapid adaptation frequency” in the different lof mutants. Twenty mutant strains of each of *ΔMohog1*, *ΔMopbs2*, *ΔMossk2*, *ΔMossk1*, and *ΔMoypd1* were grown continuously on solid complete medium (CM) including 1 M KCl or 1.5 M sorbitol as stress-inducing agents. Several adapted strains arose out of the lof mutants in each plate, being able to grow much faster under the stress condition. The adapted strains were then transferred onto CM without stressors for the following two weeks. Subsequently, we transferred the colonies onto repeated stress medium to investigate in which strains the “adaptation” is stable and identified the stably adapted strains (Additional file 3: Figure S2). Notably, we were able to identify more adapted strains from *ΔMopbs2* and *ΔMoypd1* under salt stress than under sorbitol stress. By contrast, we obtained more adapted strains under sorbitol stress than under salt stress from *ΔMossk2* and *ΔMossk1* (Additional file 3: Figure S2).

### Osmoregulation is permanently restored in the adapted mutants

**Fig. 3:**
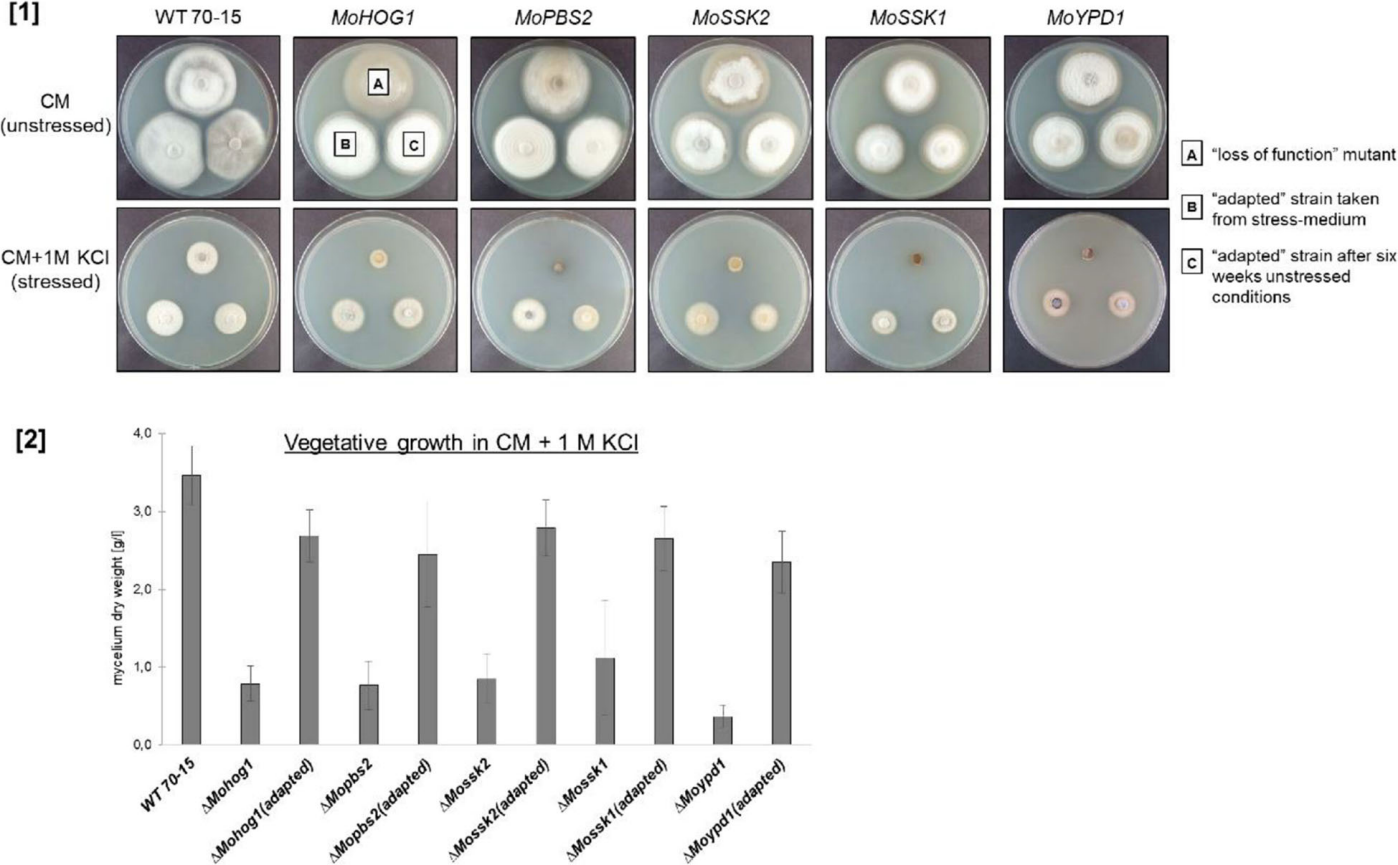
[1] Stable reestablishment of osmoregulation in adapted strains. Vegetative growth of the *Magnaporthe oryzae* wildtype strain, mutants with inactivated components of the HOG signaling cascade and the “adapted” strains upon salt stress. The fungal colonies were grown on CM (upper row) and CM including 1 M KCl (lower row) for 7 d at 26 °C. A, B and C are representative for each plate. A = lof mutant, B = “adapted” strain taken directly from CM inclusive 1 M KCl (“adaptation” conditions), and C = “adapted” strain after six weeks of preincubation on CM (to have long-term unstressed conditions before this assay). In the case of the wildtype strain (left), the three cultures on each test plate used in the assays were from the *M. oryzae* wildtype strain 70–15 cultivated in an equal manner. [2] Mycelium dry weight of the *Magnaporthe oryzae* wildtype strain, mutants with inactivated components of the HOG signaling cascade and the “adapted” strains after growth in liquid culture upon KCl stress. The fungal colonies were grown in 250 ml CM including 1 M KCl for 6 d at 26 °C and 120 rpm. Error bars represent the standard deviation of three biological replicates of each strain

The strains *ΔMohog1(adapted)*, *ΔMopbs2(adapted)*, *ΔMossk2(adapted)*, *ΔMossk1(adapted)* and *ΔMoypd1(adapted)* displayed significant differences in growth speed on salt stress compared to their “parent-strains,” the lof mutants *ΔMohog1*, *ΔMopbs2*, *ΔMossk2*, *ΔMossk1* and *ΔMoypd1* (Fig. 3 [1]). All lof mutants were strongly sensitive towards 1 M KCl stress, whereas all the adapted strains were less sensitive, similar to the wildtype strain (Fig. 3 [1], lower row, colonies [A]). Particular attention has to be paid to the finding that adapted strains, which were pre-cultivated on normal CM (unstressed conditions) and then transferred back to the repeated salt stress, were found to grow as fast as those taken directly from stress conditions and, thus, being able to adapt to KCl stress immediately (Fig. 3 [1], colonies [C]). In conclusion, the mutations/alterations within *ΔMohog1(adapted)*, *ΔMopbs2(adapted)*, *ΔMossk2(adapted)*, *ΔMossk1(adapted)* and *ΔMoypd1(adapted)* appear to be stable. Similar results to the growth assays on solid media could be observed in liquid cultures upon KCl stress (Fig. 3 [2]) and sorbitol stress (Additional file 4: Figure S3).

### Glycerol is the major compatible solute produced by the adapted strains after salt shock

**Fig. 4:**
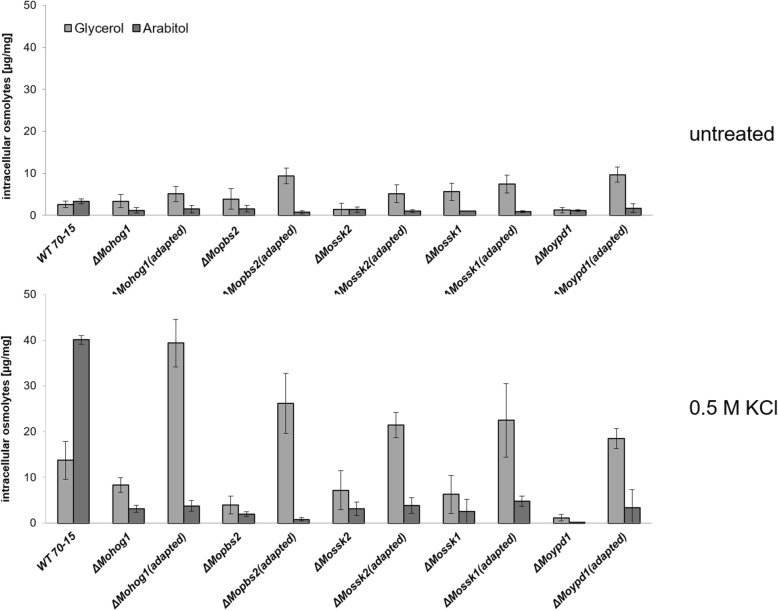
Compatible solute production in the *Magnaporthe oryzae* wildtype strain (WT 70–15), the lof mutants and the “adapted” strains after osmotic shock. Compatible solute accumulation (glycerol and arabitol) was assayed in mycelium after 7 h salt stress. The mycelium was grown for 72 h in CM (2% glucose) before being shocked with 0.5 M KCl for an additional 7 h. Carbohydrates were extracted and quantified by liquid chromatography. Error bars represent the standard deviation of three biological replicates of each strain

Intracellular production of compatible solutes was determined by high-performance anion-exchange chromatography with pulsed amperometric detection (HPAEC-PAD) and compared to compatible solute production of the lof mutants and the wildtype strain to further investigate the phenomenon of restored osmoregulation in *ΔMohog1(adapted)*, *ΔMopbs2(adapted)*, *ΔMossk2(adapted)*, *ΔMossk1(adapted)* and *ΔMoypd1(adapted)*. Hyperosmotic shock was imposed by 0.5 M KCl stress towards the fungal strains, and the intracellular levels of the major osmolytes mannitol, trehalose, arabitol and glycerol were determined. No increases in the mannitol and trehalose levels were detected in the lof mutants and the adapted strains after osmotic shock (data not shown). A slight increase in the mannitol level was detected and no significant change for the trehalose level of the wildtype strain (data not shown). Similar to data from [20], arabitol was found to be the major intracellular compatible solute produced by the wildtype strain after osmotic shock (Fig. 4).

By contrast, the lof mutants *ΔMohog1*, *ΔMopbs2*, *ΔMossk2*, *ΔMossk1* and *ΔMoypd1* were not able to produce either arabitol or glycerol in significant amounts. Interestingly, it was found that all the adapted strains *ΔMohog1(adapted)*, *ΔMopbs2(adapted)*, *ΔMossk2(adapted)*, *ΔMossk1(adapted)* and *ΔMoypd1(adapted)* responded to hyperosmotic stress by accumulating high amounts of glycerol rather than arabitol (Fig. 4). Based on these observations, we conclude that glycerol may somehow compensate for the lack of arabitol upon salt stress in the adapted strains.

### Fludioxonil susceptibility is restored in the adapted mutants

**Fig. 5:**
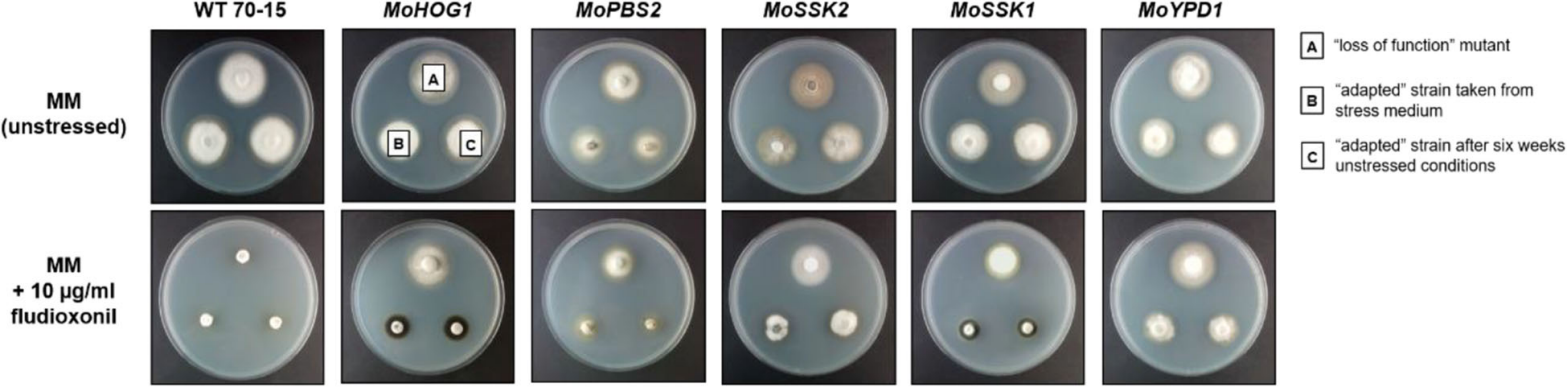
Stable vegetative growth of the Magnaporthe oryzae wildtype strain (WT 70–15), mutants with inactivated components of the HOG signaling cascade and the “adapted” strains upon fludioxonil treatment. The fungal colonies were grown on minimal medium (MM, upper row) and on MM including 10 μg/ml fludioxonil (lower row) for 7 d at 26 °C. A, B and C are representative for each plate. A = lof mutant, B = “adapted” strain taken directly from CM including 1 M KCl (“adaptation” conditions) and C = “adapted” strain after six weeks of preincubation on CM (to have long-term unstressed conditions before this assay to show the memory effect). In the case of the wildtype strain (left), the three cultures on each test plate used in the assays were from the *M. oryzae* wildtype strain 70–15 cultivated in an equal manner

Vegetative growth assays were conducted using minimal medium (MM) and MM including 10 μg/ml fludioxonil to further investigate whether fludioxonil-susceptibility and not only osmoregulation is restored in *ΔMohog1(adapted)*, *ΔMopbs2(adapted)*, *ΔMossk2(adapted)*, *ΔMossk1(adapted)* and *ΔMoypd1(adapted)* (Fig. 5).

Apart from the osmoregulation capacity, fludioxonil sensitivity was reconstituted in the adapted strains. The HOG pathway lof mutants were resistant towards the fungicide (Fig. 5, colonies [A], lower row), whereas all adapted strains were susceptible, but not quite as strongly as the wildtype strain (Fig. 5, colonies [B], lower row). Similar to the findings concerning salt stress presented previously in Fig. 3, the adapted strains, which were pre-cultivated in unstressed conditions for a long time, showed the same phenotype as the adapted strains taken directly from stress medium (Fig. 5, colonies [C], lower row).

### Fludioxonil sensitivity in the adapted strains is not dependent on the production of arabitol or glycerol

**Fig. 6:**
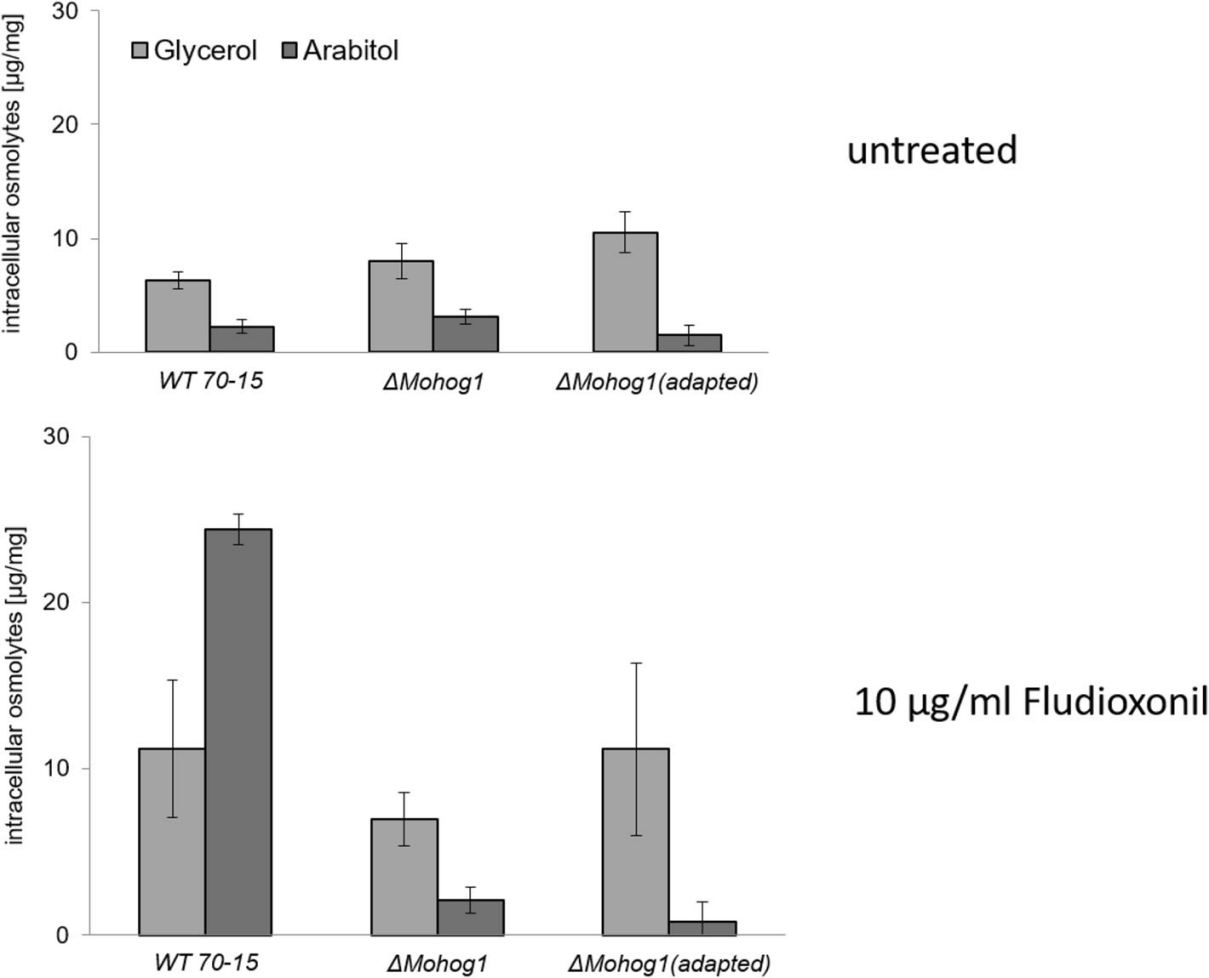
Compatible solute production in the wildtype strain, *ΔMohog1* and *ΔMohog1(adapted)* upon fludioxonil treatment. Compatible solute accumulation (glycerol and arabitol) was assayed in the mycelium of the wildtype strain, *ΔMohog1* and *ΔMohog1(adapted)* after 7 h treatment with 10 μg/ml fludioxonil. The mycelium was grown for 72 h in CM (2% glucose) before being shocked with 10 μg/ml fludioxonil for an additional 7 h. Carbohydrates were extracted and quantified by liquid chromatography. Error bars represent the standard deviation of three biological replicates of each strain

Fludioxonil induces a hyperactivation of the HOG pathway and, thus, a continuous stress response towards high osmolarity. We found, by using HPAEC-PAD analytics, that fludioxonil treatment resulted in the production of arabitol and glycerol in the wildtype strain (Fig. 6). We conducted the experiments with the lof mutants and the adapted strains *ΔMohog1(adapted)*, *ΔMopbs2(adapted)*, *ΔMossk2(adapted)*, *ΔMossk1(adapted)* and *ΔMoypd1(adapted)* to check whether fludioxonil-dependent production of the compatible solutes was altered in the adapted strain. The fungal cultures were grown in liquid CM (2% glucose) and stressed with 10 μg/ml fludioxonil. As expected, we did not find increased compatible solute production upon fludioxonil-treatment in the lof mutants. Exemplarily, we present the data for *ΔMohog1* (Fig. 6).

Interestingly, no increase of arabitol or glycerol production was detectable in the fludioxonil-susceptible adapted strains in the presence of the fungicide (Fig. 6). It has to be pointed out that the metabolic response of the adapted strains after fludioxonil treatment is different compared to the compatible solute production we observed upon KCl treatment (Fig. 4). All the adapted strains *ΔMohog1(adapted)*, *ΔMopbs2(adapted)*, *ΔMossk2(adapted)*, *ΔMossk1(adapted)* and *ΔMoypd1(adapted)* responded to salt stress by accumulating high amounts of glycerol, whereas this is not the case under fludioxonil stress. In conclusion, fludioxonil sensitivity in the adapted strains does not appear to be dependent on compatible solute production.

### Reestablished osmoregulation does not complement reduced virulence of the lof mutants

The lof mutants of the HOG pathway in *M. oryzae* were found to be reduced in virulence towards rice plants compared to the wildtype strain. Interestingly, *ΔMohog1(adapted)*, *ΔMopbs2(adapted)*, *ΔMossk2(adapted)* and *ΔMossk1(adapted)* were not found to be as virulent as the wildtype strain, and rather less virulent than the lof mutants (Additional file 5: Figure S4). We were not able to conduct the pathogenicity assays regarding *ΔMoypd1(adapted)*, since the mutant failed to produce conidia exactly like *ΔMoypd1* [21].

### There are no relevant structural variations on DNA-level in the genomes of adapted strains

We had a deeper look at the genomes of *ΔMohog1* compared to *ΔMohog1(adapted)* in order to find out the cause of that rapid adaptation in the adapted strains. We also added data from the genome sequencing of *ΔMopbs2(adapted)* to strengthen the analysis and narrow down the outcome of putative candidate genes showing structural variations in the adapted strains. Single nucleotide variations (SNVs) and short indels were detected for *ΔMohog1(adapted), ΔMopbs2(adapted)* and *ΔMohog1* in comparison with the reference sequence of the wildtype strain. The resulting variants were further annotated based on their chromosomal location and biological effects, such as synonymous/non-synonymous single-nucleotide polymorphisms (SNPs), upstream/downstream, untranslated regions (UTRs) and intergenic regions (Additional file 1: SVs summary). The ratio of transition and transversion was also calculated for single nucleotide variation. Over 80% of all SNPs detected were found to be located outside exons and a significant enrichment in regions adjacent to exons and UTRs was detected. Furthermore, in silico protein modelling suggested that several non-synonymous SNPs are probably direct targets of selection, as they lead to amino acid replacements in functionally important sites of proteins. Hence, the structural variation discovery analysis of small-scale (< 20 bp) and large-scale variations (> 20 bp) such as frameshift, stop codon insertion resulted in a list of three genes (five putative gene variants) in the overlap of *ΔMohog1(adapted)* and *ΔMopbs2(adapted)*, but none of them leads to a protein effect. The variations are only transitions not changing the amino acid composition of the corresponding proteins. Furthermore, we checked the homologous gene loci of the known yeast suppressor mutation genes *RSG1* (*RHB1*) [25], *SOO1* [26], *SGD1* [27] and *PMK1* (*KSS1*) [28] intensively. Within all these loci, we could not identify structural variations in the genome of the adapted strains *ΔMohog1(adapted)* and *ΔMopbs2(adapted)*.

Since regulatory gene elements, such as promoters, are of prior interest regarding their direct influence on gene expression alteration, we decided to investigate the presence of genetic structural variations in the putative promoter regions of all annotated genes in the *M. oryzae* genome. The promoter region was defined as the region on the genomic DNA 1500 bp upstream of each annotated gene start codon. We performed a structural variation discovery analysis of small-scale (< 20 bp) and large-scale variations (> 20 bp) resulting again in no significant variations in the overlap of the promoter regions of *ΔMohog1(adapted)* and *ΔMopbs2(adapted)* (Additional file 6: Figure S5 [A]). We considered here only insertions and deletions as a “polymorphism type” and insertions, truncations and frame-shifts as a “protein effect.”

### Differential transcriptomic profiles in the adapted strains in response to osmotic stress

**Fig. 7:**
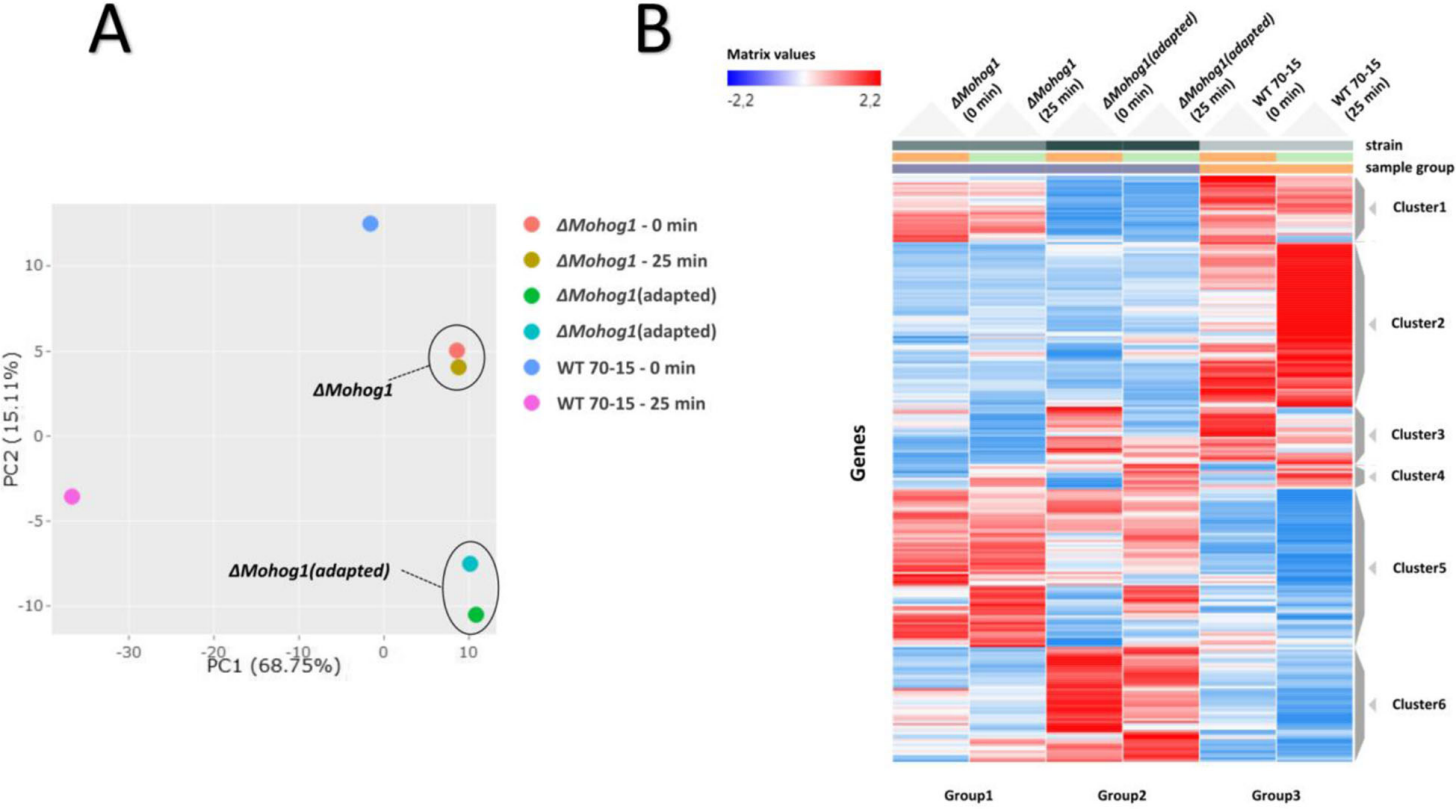
RNA-sequencing (Seq) data processing. **a** Principal component analysis results. The figure displays a three-dimensional scatter plot of the first three principal components (PCs) of the data. Each point represents an RNA-Seq sample. Samples with similar gene expression profiles are clustered together. Sample groups are indicated by using different colors as indicated in the legend provided. **b** Heat map of transcriptional regulation patterns of the wildtype strain, *ΔMohog1* and *ΔMohog1(adapted)* after 25 min salt stress (Clustergrammer analysis). The figure contains a heat map displaying the expression profile of the top 500 differential expression genes (DEGs) for each sample in the RNA-Seq dataset. The set of mutant strains *ΔMohog1* and *ΔMohog1(adapted)* prior and after salt stress was compared to wildtype strain 70–15 (indicated in the figure as sample group) for perturbation contrast. Each row of the heat map represents a gene, each column represents a sample and each cell displays normalized gene expression values

Next-generation sequencing analysis of RNA samples from the wildtype strain, *ΔMohog1* and *ΔMohog1(adapted)* before and after 25 min salt stress (0.5 M KCl) should present insights into transcriptional changes which may be responsible for the adaptation phenomenon observed (methods for cultivation before RNA-isolation, see [29]). A principal component analysis was performed to characterize the relationship between the strains analyzed. The processed transcriptome data of *ΔMohog1* (untreated), *ΔMohog1* (25 min 0.5 M KCl-stress), *ΔMohog1(adapted)* (untreated) and *ΔMohog1(adapted)* (25 min 0.5 M KCl-stress) appears to form distinct clusters within each sample group of strain samples investigated (Fig. 7a).

Since the principal component analysis revealed distinct differences on transcriptomes of corresponding strains, we decided to follow up by clustering the genes according to their expression followed by construction of a co-expression network. In the clustering analysis, represented by a heat map visualization, the fact that the transcript value is strongly different in *ΔMohog1(adapted)* compared to the WT 70–15 and *ΔMohog1*, even in untreated conditions, is clearly visible (Fig. 7b). Cluster 6 is significantly exclusively upregulated and cluster 1 is harboring exclusively down-regulated genes in *ΔMohog1(adapted)*, whereas exclusively up-regulated genes in the WT 70–15 could be found in cluster 2 (Fig. 7b).

### Glycerol metabolism-associated genes are affected in the adapted strains

**Fig. 8:**
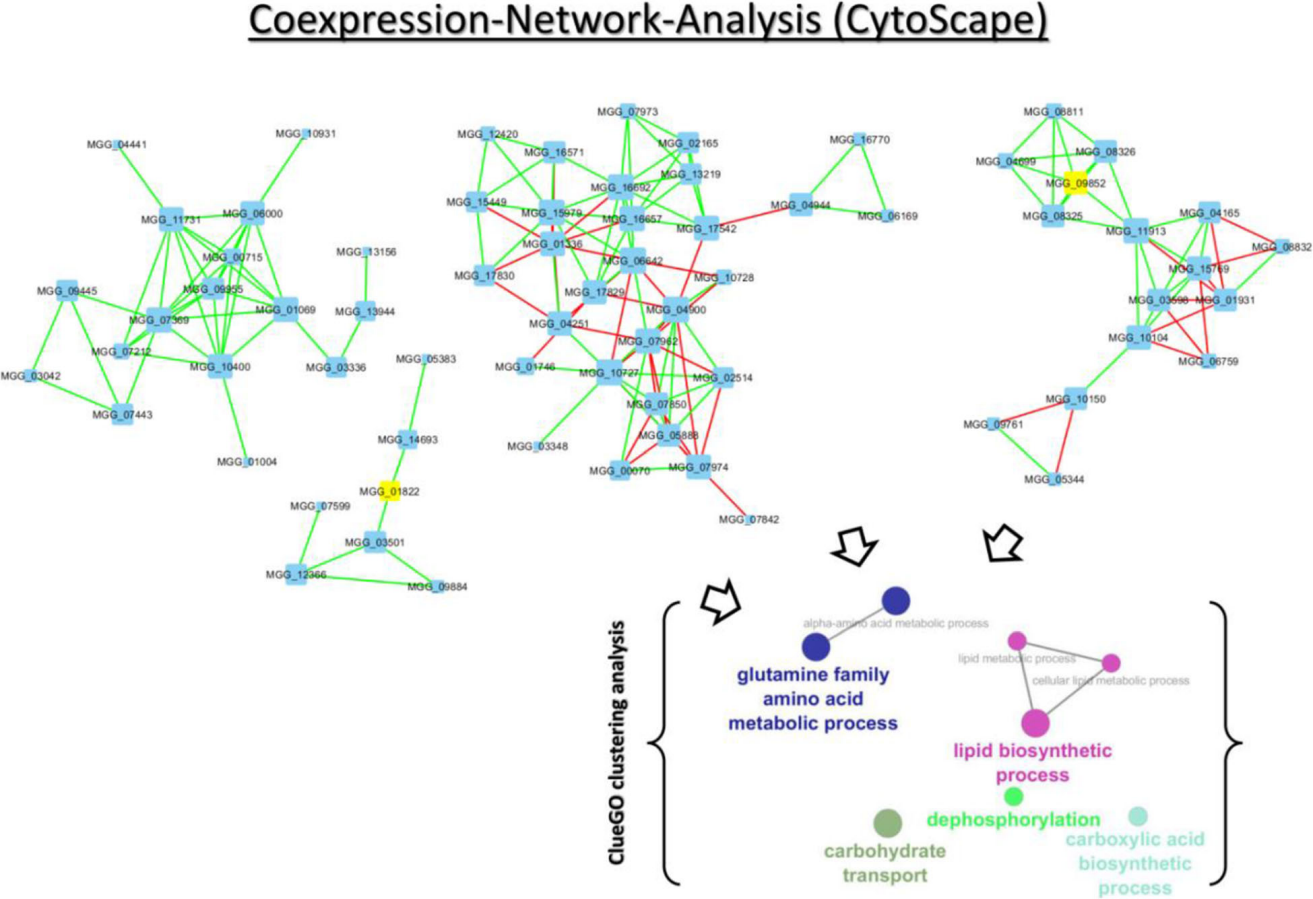
Network visualization and analysis obtained by the Cytoscape/ClueGO clustering pipeline. The size of the nodes reflects the statistical significance of each term. Subnetworks of genes that are highly connected (network cluster) in a co-expression network are usually involved in similar biological functions. The cluster analysis of the network was performed to find groups of genes that may be acting in a coordinated manner. A gene ontology (GO) enrichment analysis was conducted using ClueGO to determine the biological functions associated to them to investigate if a group of genes are functionally related. The name of each group is given by the most significant term of the group. This analysis was performed over the whole network

To investigate, whether the groups of differentially expressed genes (DEGs) are functionally related, we performed a gene ontology (GO) enrichment analysis to determine the biological functions associated to them. Generally, this approach helps to highlight groups of genes with coherent biological functions that are presumably acting in coordination in response to salt stress. The clustering analysis was employed over the whole gene co-expression network and in a selected subset or cluster of interest. The result indicated that there were several significantly enriched terms of DEGs, from which the most representative are “lipid biosynthetic process,” “glutamine family amino acid metabolic process,” “carbohydrate transport,” “dephosphorylation” and “carboxylic acid biosynthetic process” (Fig. 8).

This enrichment analysis suggests that the highly connected genes from the largest cluster in the network are involved in the regulation of complementary processes triggered by salt stress.

In order to follow up the results of glycerol-production in the adapted strains, we further investigated most of the genes potentially contributing to the production, metabolism or transport of glycerol. However, we checked these genes presented resulting in a list of homologous genes potentially related to the production, metabolism or transport of glycerol from a database- and literature-based approach (Table 1).

**Table 1:**
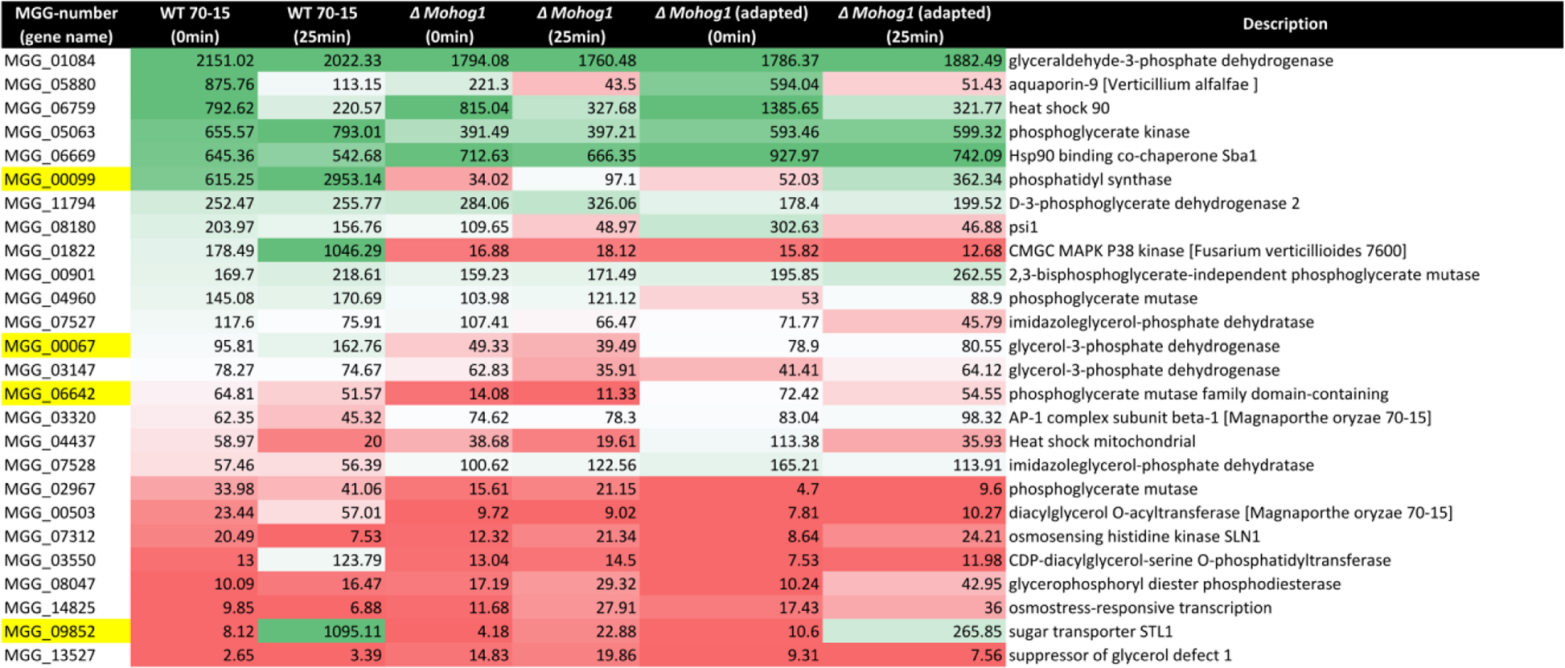
Glycerol biosynthesis and osmotic stress response-related genes being differentially expressed across the strains analyzed

The table shows the glycerol biosynthesis and osmotic stress response-related genes retrieved being differentially expressed across the strains analyzed. The colors used in the table indicate the percentiles projected on the entire amounts of the transcripts counted (green stands for 90%, white for 50% and red for 10%)

The analysis resulted in a set of candidate genes which were found to be upregulated in both the salt stress samples of the *ΔMohog1(adapted)* and the wildtype strain, whereas these genes were not regulated in the lof mutant *ΔMohog1* (Table 1, yellow marked). Among these candidates, we identified genes encoding the glycerol H^+^-symporter MoSlt1p (MGG_09852), one phosphoglycerate mutase (MGG_06642,), one glycerol-3-phosphate dehydrogenase (MGG_00067 (MoGpd1p)) and one phosphatidyl synthase (MGG_00099 (MoHad1p)).

### The HOG pathway is not responsible for adaptation in *ΔMohog1(adapted)*

**Fig. 9:**
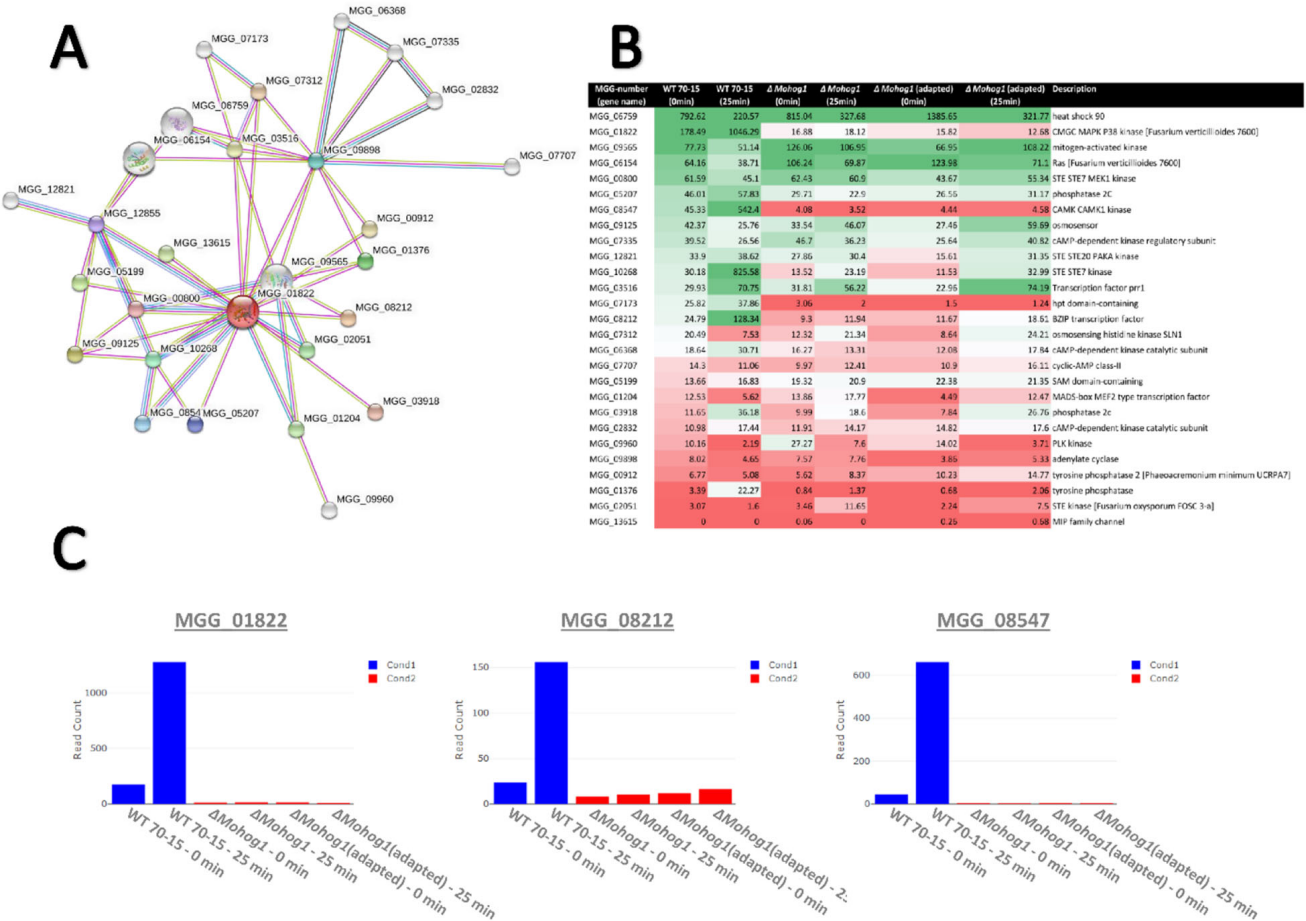
Interaction network analyzed by the STRING website and gene expression profiles of *HOG1*-related genes across the strains analyzed in this study. **a** STRING network visualization showing 30 proteins known to interact with Hog1p in yeast. With a confidence cutoff of 0.9, the resulting network contains 48 functional associations between 30 of the proteins. **b** Expression profiles of DE genes obtained from the STRING analysis across the strains analyzed. **c** Expression profiles of candidate genes derived from the transcriptome analysis of the strains selected

We searched for possible interactions between MoHog1p and other osmotic stress responsive or associative gene products using the SMART website to find links between putative interaction partners of the MAPK MoHog1p in the adapted strains. The genes identified were finally used to inspect their expression patterns within our set of DE genes (STRING analysis, Fig. 9a).

As expected, the transcript level of the responsive genes belonging to HOG pathway were found to be highly upregulated in case of the wildtype strain in response to osmotic stress (Fig. 9b). Thus, among the genes with the most abundant transcripts were MGG_01822, MGG_08212 and MGG_08547 encoding *HOG1*, a BZIP transcription factor and *CAMK1* kinase, respectively (Fig. 9b, c. Meanwhile, none of the *HOG1*-associated genes, except for MGG_06759 encoding a heat shock protein, were transcriptionally active in *ΔMohog1* and in *ΔMohog1(adapted)* (Fig. 9b). These results have been validated by means of qPCR (Additional file 7: Figure S6). That leads to the conclusion that other mechanisms operating independently/outside of the HOG pathway may be responsible for the phenotype observed in the adapted strains.

## Discussion

A central focus in biology is evolution and understanding the processes facilitating or preventing it. Research in this field has to integrate information at the organism level with the sequence level, transcriptional control, epigenetic modulation or cellular differentiation [3]. Despite their complexity, biological networks have an inherent similarity: They are modular with a design that can be separated into single units which perform almost independently. Little is known about the evolutionary origin of these properties. It is of great interest to better understand how signaling pathways can be modified to generate new biochemical reactions or new morphologies [30].. How are new components integrated into existing signaling pathways and how evolve the pathways themselves? For example, a genome sequence analysis in yeast has identified healthy individuals even though carrying disease-related mutations. A second genomic mutation that can compensate for the detrimental effects of the disease allele may serve as possible explanation, that phenomenon is known as suppression [31]. Most of such suppression interactions are reported between genes that are annotated to the same biological process and thus have related functions [32–34]. This means that suppression appears to be caused by mutations in genes that are functionally similar to the query [8]. Suppression interactions are generally subdivided into two classes: Genomic suppressors and dosage suppressors. The first ones are mutations in the genome that bypass a mutant phenotype, and in the second ones, alteration of gene expression such as overexpression of a suppressor gene rescues a mutant phenotype [31]. Within this study, we found a suitable system to study rapid evolutionary adaptation processes in eukaryotic microorganisms, more exactly in fungal individuals. The physiological role of the HOG pathway is to orchestrate the adaptation of cells to increased osmolarity of the surrounding medium [35]. The lof mutants *ΔMohog1*, *ΔMopbs2*, *ΔMossk2*, *ΔMossk1* and *ΔMoypd1* in *M. oryzae* are strongly impaired in osmoregulation and are resistant to the fungicide fludioxonil [21]. We present that long-term cultivation upon high osmolarity resulted in stable individuals being restored in osmoregulation outgrowing from each of these lof mutants. Interestingly, the major compatible solute produced by these adapted strains upon salt stress is glycerol, whereas it is known to be arabitol in the wildtype strain (Fig. 4). In order to unravel this phenomenon, DNA- and RNA-Seq analysis was performed in the adapted strains compared to their lof-parent strains. The structural variation discovery analysis leading to protein effect (such as frameshift, stop codon insertion) resulted in only transitions not changing the amino acid composition of the corresponding proteins. Structural variations observed on the genomic landscape may generally have an impact (negative or positive) on the gene expression, which, in turn, may explain the adaptive phenotype observed. We have to distinguish between structural genomic variations seen in exons or CDS and those located in the regulatory elements of the genes (such as promoters, enhancers and terminators). The latter may cause variations in the gene expression, which necessitates the comparative analysis of SNV analysis data with RNA-Seq data (DEGs). On the other hand, the SNV located in CDS and exons is not expected to be reflected in the DEG. The encoded products may have an impact on the secondary and tertiary structure, caused by amino acid replacements, which may lead to reduced or, in some cases, enhanced functionality of the enzyme/protein. We have not been able to identify structural genomic variations in the regulatory elements of the genes so far (Additional file 6: Figure S5 [A]). Of course, we have to increase the number of adapted strains from which we will sequence the genomes to get a higher amount of reliable data to analyze genomic changes in more detail. On transcriptome-level of the adapted strains, the interaction network conducted by SMART analysis revealed that none of the *HOG1*-associated genes, except for one heat shock protein, were transcriptionally active after salt stress in *ΔMohog1* and *ΔMohog1(adapted)* in contrast to the *MoWT* (Fig. 9). This leads to the conclusion that other mechanisms operating independently of the HOG pathway may be responsible for the phenotype observed in the adapted strains. We found that set of candidate genes potentially contributing to the production, metabolism or transport of glycerol, which is significantly upregulated after salt stress in *ΔMohog1(adapted)* and the wildtype strain, whereas these genes were not regulated in the lof mutant *ΔMohog1* (Table 1). These results fit well to the increased production of the compatible solute glycerol in the adapted strains in response to salt stress (Fig. 4). The phenomenon of the adapted strains *ΔMohog1(adapted)*, *ΔMopbs2(adapted)*, *ΔMossk2(adapted)*, *ΔMossk1(adapted)* and *ΔMoypd1(adapted)* is different to the yeast suppressor mutants regarding the genes *RSG1* (*RHB1*) [25], *SOO1* [26], *SGD1* [27] and *PMK1* (*KSS1*) [28]. Exemplarily, some Hog1p-dependent osmotic stress-induced gene expression patterns in *S. cerevisiae Δhog1* mutants could be synthetically rewired under the control of Fus3p/Kss1p MAPKs (PMK1 homologues) [36]. Furthermore, alteration of only one amino acid in Kss1p suppresses the osmosensitive growth phenotype of the *Δhog1* deletion mutant [28]. Moreover, it is known that overexpression of the *GPD1*-gene partly rescued the osmosensitive phenotype [37] and the gene *SOO1* complements the temperature-dependent osmosensitivity of *Δhog1* [26]. Overexpression of *SGD1* partially suppresses the osmosensitivity of *Δpbs2* and *Δhog1* mutants [27]. In conclusion, the loss of one gene may be compensated by another gene with overlapping functions and expression patterns, as reported for several mutants in a range of model organisms [38]. Thus, disorder of a particular gene’s function in a network may alter the expression of other genes within the same network, thereby maintaining cellular homeostasis [9, 39]. Gene deletion in yeast may result in mutations in one or more genes modulating the pathway affected, thereby, partially or fully rescuing the final outcome [40]. However, all of the statements above are based on results found by using the tractable yeast knockout collection and analysis thereof [40], and all findings have only been made in mapping and interpreting the growth rate as a proxy for fitness [8]. By contrast, the current study has the potential to open the door for many research groups worldwide working on the HOG pathway in different eukaryotic model organisms to study epigenetic and evolutionary adaptation mechanisms from a different point of view. It is of the highest scientific interest to obtain such adapted strains in other model organisms apart from *M. oryzae*. The yeasts *S. cerevisiae* and *Schizosaccharomyces pombe* [41] are, for example, model organisms from which to acquire adapted strains from HOG pathway lof mutants. Further candidates will be the opportunistic human fungal pathogens *Candida albicans* [42], *Aspergillus fumigatus* [43] or the basidiomycetous human fungal pathogen *Cryptococcus neoformans* [44]. The ascomycetes *Aspergillus nidulans* [45, 46], *Botrytis cinerea* [47], *Fusarium graminearum* [48] and *Neurospora crassa* [49], or even the plant pathogenic basidiomycete *Ustilago maydis* [50] will also be promising candidates, since the HOG pathway has been studied intensively in all of these fungi*.*

## Conclusion

We found the filamentous rice blast fungus *Magnaporthe oryzae* to rapidly reestablish signal transduction required for osmoregulation in independent osmosensitive “loss-of-function” (lof)-mutants of the High Osmolarity Glycerol (HOG)-pathway upon exposure to salt stress. Adaptation resulted in stable mutants of the model organism being reestablished in osmoregulation arising as individuals outgrowing from the salt-sensitive mutants. These findings lead to the hypothesis that stable adaptation-events under continuously environmental evolutionary pressure enable *Magnaporthe oryzae* to rapidly restore or modify entire signaling networks.

This study provides evidence, that *M. oryzae* is a suitable model organism to study rapid evolutionary processes in eukaryotes. Furthermore, it will open a door for the scientific community to study this topic since the HOG pathway is worked on intensively in many different eukaryotic model organisms.

## Methods

All data generated or analyzed during this study are included in this published article or deposited at the NCBI GenBank SRA database under accession number PRJNA559166 and Bioproject number 559166.

### Strains, culture/growth conditions

The fungal strain used in this study was *Magnaporthe oryzae* (*M. oryzae* 70–15 strain (*MoWT*), Fungal Genetics Stock Center (FGSC)) and the lof mutants of the HOG pathway generated previously [21]. The strains were grown at 26 °C on CM (pH 6.5, 2% agar, containing per liter: 10 g glucose, 1 g yeast extract, 2 g peptone, 1 g casamino acids, 50 mL nitrate salt solution (containing per liter: 120 g NaNO_3_, 10.4 g KCl, 30.4 g KH_2_PO_4_, 10.4 g MgSO_4_ × 7 H_2_O) and 1 mL of a trace element solution (containing per liter: 22 g ZnSO_4_ × 7 H_2_O, 11 g H_3_BO_3_, 5 g MnCl_2_ × 4 H_2_O, 5 g FeSO_4_ × 7 H_2_O, 1.7 g CoCl_2_ × 6 H_2_O, 1.6 g CuSO_4_ × 5 H_2_O, 1.5 g Na_2_MoO_4_ × 2 H_2_O, 50 g Na_2_EDTA, pH 6.5 adjusted by 1 M KOH). The MM (pH 6.5) contains per liter: 1 g glucose, 0.25 mL of a 0.01% biotin solution, 50 mL nitrate salt solution, 1 mL of a trace element solution and 1 mL of a 1% thiamine dichloride solution.

All chemicals used were *p.a*. quality unless stated otherwise.

### Vegetative growth assays

The antifungal activity and stress tolerance of vegetative growth on agar plates were conducted according to [24]. In order to verify the results, we determined the mycelium dry weight of the fungal colonies grown in liquid culture. The fungal colonies were grown in 250 ml CM including sorbitol or KCl for 6 d at 26 °C and 120 rpm.

### Empirical investigation of the “adaptation” frequency

In order to determine the adaptation in *M. oryzae*, we conducted a combination of different vegetative growth assays to generate adapted mutant strains to characterize the “adaption frequency.” We used a panel of 11-day-old lof mutant strains of each of *ΔMohog1*, *ΔMopbs2*, *ΔMossk2*, *ΔMossk1*, *ΔMoypd1*, *ΔMohik1* and *ΔMosln1* to inoculate solid CM including 1 M KCl or 1.5 M sorbitol as stress-inducers. The most suitable concentration ranges of the individually selected stressors was chosen based on our previous work in the osmoregulation pathway [21, 24]: The mutants should be clearly restricted in growth but still being able to grow. All fungal cultures have been incubated at 26 °C and a light/dark rhythm of 16/8 h and have been regularly transferred to fresh medium. After 8–12 weeks, we selected all outgrowing mycelium parts (individuals) from this experimental setup and put them on normal CM without stressors for the following two weeks. We then put the colonies on repeated stress medium to investigate in which strains the “adaptation” is stable.

### Plant infection assays

The plant infection assays were carried out as described previously [24].

### HPAEC-PAD analysis to quantify compatible solute production

Osmolytes (sugar alcohols, monosaccharides and disaccharides) were analyzed using HPAEC. They were separated as anions under high alkaline conditions (pH > 12), coupled with PAD. Dried cell extracts were solved (sonificated) in 1 ml sterilized, double-distilled water and centrifuged (4 °C, 13,000 rpm). All the samples were filtrated through a 0.2 μm filter. The injection volume was 10 μl. The HPAEC-PAD analysis was performed on a Shimadzu LC system equipped with two LC-10Ai pumps, a DGU-20A degassing unit, a SIL-10Ai autosampler, a CBM-20A controller and a CTO-20 AC column oven. An analysis anion-exchange column of CarboPac MA1 (4 × 250 mm) in combination with a guard column of CarboPac MA1 (4 × 50 mm) at 20 °C was used. A Dionex ED40 Electrochemical detector was used for the detection of carbohydrates and sugar alcohols in pulsed amperometric mode through standard quadruple waveform (t = 0–0.40 s, *p* = 1.00 V; t = 0.41–0.42 s, *p* = − 2.00 V; t = 0.43 s, *p* = 6.00 V; t = 0.44–0.50s, *p* = − 1.00 V). The eluent was prepared as a mobile phase which consisted of a 480 mM NaOH solution. Isocratic elution was performed at 0.4 ml/min. Aqueous sodium hydroxide (50%, w/w) was obtained from Merck, Germany. Standards were purchased from Sigma-Aldrich (Germany) and Carl Roth (Germany). Solutions were prepared in double-distilled water with a concentration of 10 mg/L.

### Bioinformatics and data visualization

The DEBrowser recently published was used for data assessment. Firstly, the filtering of the genes was performed. The maximum count for each gene across all samples less than a threshold set to 10 was chosen as the filtering criterium. The application provides a rich and interactive web-based graphical user interface built on R’s shiny infrastructure. The novel method, called “Median Ratio Normalization,” which provides the lower number of false discoveries was used for the normalization of data prior to processing to heat maps and other plots.

The interactive heat map was generated using Clustergrammer [51] which is freely available at http://amp.pharm.mssm.edu/clustergrammer/. Clustergrammer is a web-based tool for visualizing and analyzing high-dimensional data as interactive and hierarchically clustered heat maps. It is commonly used to explore the similarity between samples in an RNA-Seq dataset. In addition to identifying clusters of samples, it also allows one to identify the genes which contribute to the clustering. Prior to displaying the heat map, the raw gene counts were normalized using the logCPM method, filtered by selecting the 500 genes with the most variable expression and, finally, transformed using the Z-score method.

### Transcriptome sequencing and differential gene expression

Transcriptome differences in response to salt stress were determined by RNA-Seq using next-generation sequencing. Consequently, the RNA of the strains *M. oryzae* 70–15, *ΔMohog1* and *ΔMohog1(adapted)* was isolated using the RNeasy® Plant Mini Kit (Qiagen). Two conditions were analyzed: Prior salt stress (0 min) and salt stress incubation after 25 min. We collected *M. oryzae* samples for the transcriptome sequencing from two different growing conditions and used three replicates each: At 0 and 25 min.

The RNA isolated was subsequently quantified using the 2100 Bioanalyzer High Sensitivity DNA kit (Agilent Technologies, Santa Clara, CA, USA) and sequenced in a 150 bp paired-end run on an Illumina HiSeq 2500. Library preparation was performed by IMSB (Mainz, Germany) using 1.5 μg of total RNA for each strain. The raw fastq reads were paired and aligned to a 70–15 strain reference sequence (assembly version MG8, http://fungi.ensembl.org/Magnaporthe_oryzae/Info/Index) using default parameters with Geneious RNA Mapper (version 11.1.5).

The Integrative Genomics Viewer ( [52, 53]) was then used to visualize the transcripts assembled. The DEGs were identified by the DEseq2 [54]. Genes with an adjustment of *p*-values by Benjamini Hochberg FDR (FDR < 0.05) and fold change values > 2.5 were considered to be differentially expressed.

Considering the processed RNA-sequencing (Seq) data, a total of 684 differentially expressed genes (DEGs) (176 up- and 508 down-regulated) were identified in the WT 70–15 vs. *∆Mohog1* + *∆Mohog1(adapted)* comparison; while 49 DEGs (23 up- and 26 down-regulated) were identified in the WT 70–15 + *∆Mohog1(adapted)* vs. *∆Mohog1* comparison. Meanwhile, a total of 16 DEGs (9 up- and 7 down-regulated) were present in the WT 70–15 (untreated) + *∆Mohog1* (untreated) + *∆Mohog1(adapted)* (untreated) vs. WT 70–15 (25 min 0.5 M KCL-stress) + *∆Mohog1* (25 min 0.5 M KCL-stress) + *∆Mohog1(adapted)* (25 min 0.5 M KCL-stress) comparison.

### RNA isolation, cDNA amplification and qRT-PCR analysis

We conducted a qPCR analysis of selected genes previously found to be strong regulated in our RNA-seq results for validation of the datasets. The *M. oryzae* cultures were grown for 96 h in CM at 26 °C and 100 rpm. Each of the cultures were separated into two samples, one mixed with 0.5 M KCl and one an untreated control. Samples for RNA isolation were taken after 25 min. The RNA was isolated from the mycelium samples and the results of transcript abundance given relative to quantification in the *MoWT* untreated control. The RNA of the *MoWT* and the mutants was isolated according to [29] using a RNeasy® plant mini Kit (Quiagen GmbH), following the manufacturer’s instructions for purification of total RNA from plants and filamentous fungi. The cDNA amplification and qRT-PCR were performed using the iScript™ One-Step RT-PCR Kit with SYBR® Green (Bio-Rad Laboratories GmbH), following the manufacturer’s instructions. We used the tubulin gene (MGG_06650) and the EF1-alpha gene (MGG MGG_03641) as reference genes (housekeeping genes) for the relative quantification of the expression ratio. Calculations were based on the relative quantification method of [55].

### SNP analysis

Sequencing reads mapped against the 70–15 reference genome (as .bam files) were used to detect structural genomic variants. Both SNVs and short indels were detected for all strains in comparison with the reference sequence of 70–15. We used the Genome Analysis ToolKit pipeline for SNP calling. The alignment (.bam) files were manually visualized by the Integrative Genomics Viewer to identify commonly associated SNPs. We used VarScan2 and Pindel for the detection of small indels [56]. We used Pindel for large indels and structural variations as it is described as working well on variations between 1 and 1000 nt. BreakDancer-predicted variants were filtered based on mapping quality greater than 25 read, read depths greater than 10 and strand level evidence (at least one read from both directions). SnpEff software was employed to analyze and annotate the resulting SNPs with the *M. oryzae* sequence and the GFF annotation file (MG8, http://fungi.ensembl.org/Magnaporthe_oryzae/Info/Index) in terms of their chromosomal location and biological effects, such as synonymous/non-snonymous SNPSs, UTRs, intergenic or upstream/downstream.

## Supplementary information

**Additional file 1:** SVs summary.

**Additional file 2: Figure S1.** Schematic presentation and verification of the *MoWT*, the lof-mutants and the adapted strains by southern hybridization within the *Magnaporthe oryzae* genome. Southern blot analysis of gene deletion/disruption mutants in *M. oryzae* with gene specific probes. Genomic DNA of *M. oryzae* strain 70–15 and the mutants was isolated and restricted with restriction enzymes. The probes which we used for hybridization with the genomic DNA of the wildtype strain and the corresponding mutant strains were always identical.

**Additional file 3: Figure S2.** Investigation of the “adaptation-frequency” in *Magnaporthe oryzae* mutants with inactivated components of the HOG signaling cascade.

**Additional file 4: Figure S3.** Mycelium dry weight of the Magnaporthe oryzae wildtype strain, mutants with inactivated components of the HOG signaling cascade and the “adapted” strains after growth in liquid culture upon sorbitol-stress. The fungal colonies were grown in 250 ml complete medium inclusive 1,5 M sorbitol for 6 d at 26 °C and 120 rpm. Error bars represent the standard deviation of three biological replicates of each strain.

**Additional file 5: Figure S4.** Pathogenicity assay of the MoWT, the lof mutants and the “adapted” strains. The plant infection assays were carried out as described in experimental procedures. The error bars represent the standard deviation of three experiments with three replicates each.

**Additional file 6: Figure S5.** VENN diagram of putative structural variations in promotor [A] and in coding sequences (CDS) [B] within the genome of *ΔMohog1, ΔMohog1(adapted)* and *ΔMopbs2(adapted)*. Numbers in the intersection regions represent overlapping SNPs among the strains. Numbers in parentheses show the corresponding relative percentage of genes harbouring the SNPs.

**Additional file 7: Figure S6.** qPCR results of selected genes. qRT-PCR analysis of putative genes in MoWT, the “lof” mutants ΔMohog1 and ΔMohog1(adapted). The *M. oryzae* cultures were grown for 96 h in CM at 26 °C and 100 rpm. Each of the cultures was separated into two samples, one mixed with 0.5 M KCl and one untreated control further grown in CM at 26 °C and 100 rpm). Samples were taken after 25 min. The RNA was isolated from the mycelium samples and the results of transcript abundance given relative to quantification in the MoWT untreated control. Three biological replicates were used of each.

## Data Availability

All data generated or analyzed during this study are included in this published article or deposited at the NCBI GenBank SRA database under accession number PRJNA559166 and Bioproject number 559166.

## Abbreviations

CM: Complete medium
DEGs: Differentially expressed genes
ERK: Extracellular signal-regulated kinase
GO: Gene ontology
HK: two-component hybrid histidine kinase
HOG: High osmolarity glycerol
HPAEC-PAD: High-performance anion-exchange chromatography with pulsed amperometric detection
lof: “Loss of function”
MAPK: Mitogen-activated protein kinase
MAPKK: MAPK kinase
MAPKKK: MAPK kinase kinase
MM: Minimal medium
PCs: Principal components
SNPs: Single-nucleotide polymorphisms
SNVs: Single nucleotide variations
UTRs: untranslated regions
WT 70–15: *Magnaporthe oryzae* wildtype strain
