# Supplementary material for: Rapid adaptation of signaling networks in the fungal pathogen Magnaporthe oryzae
Source: BMC Genomics. 2019 Oct 22;20:763. doi: 10.1186/s12864-019-6113-3 (PMC6805500; doi:10.1186/s12864-019-6113-3)
Supplement: Supplementary file 2 — Additional file 2: Figure S1. Schematic presentation and verification of the MoWT, the lof-mutants and the adapted strains by southern hybridization within the Magnaporthe oryzae genome. Southern blot analysis of gene deletion/disruption mutants in M. oryzae with gene specific probes. Genomic DNA of M. oryzae strain 70–15 and the mutants was isolated and restricted with restriction enzymes. The probes which we used for hybridization with the genomic DNA of the wildtype strain and the corresponding mutant strains were always identical. [file 12864_2019_6113_MOESM2_ESM.docx]

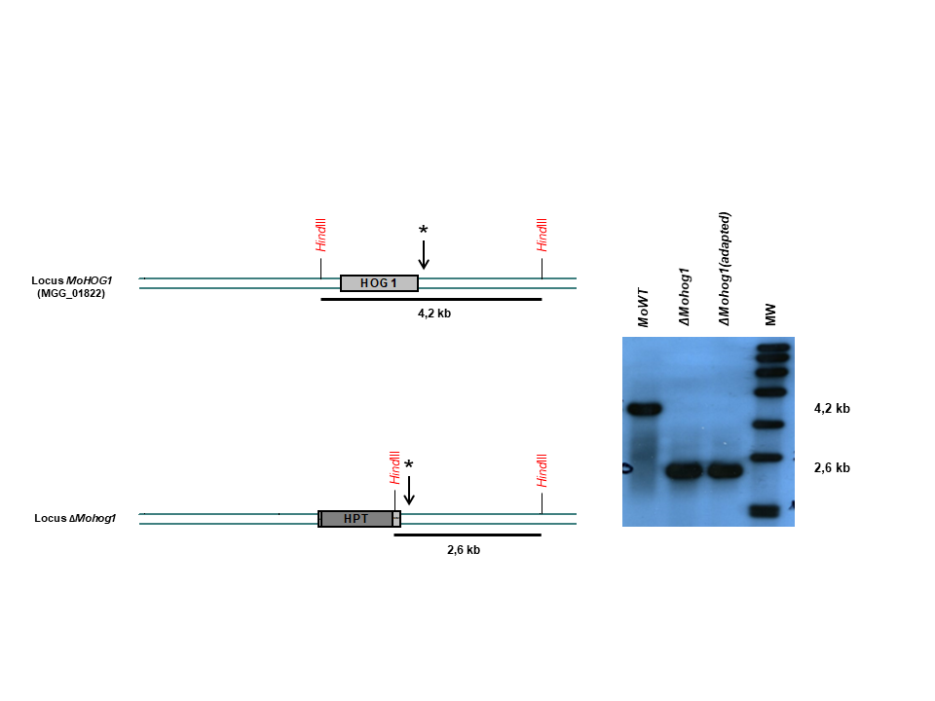


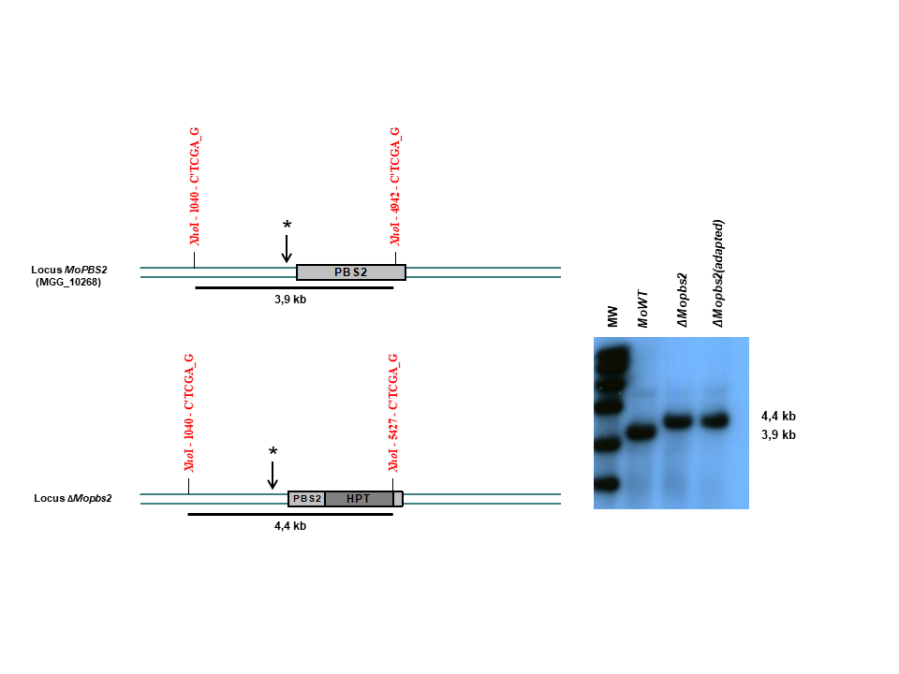


**Figure S1: Schematic presentation and verification of the *MoWT*, the lof-mutants and the adapted strains by southern hybridization within the *Magnaporthe oryzae* genome.** Southern blot analysis of gene deletion/disruption mutants in *M. oryzae* with gene specific probes. Genomic DNA of *M. oryzae* strain 70-15 and the mutants was isolated and restricted with restriction enzymes. The probes which we used for hybridization with the genomic DNA of the wildtype strain and the corresponding mutant strains were always identical.


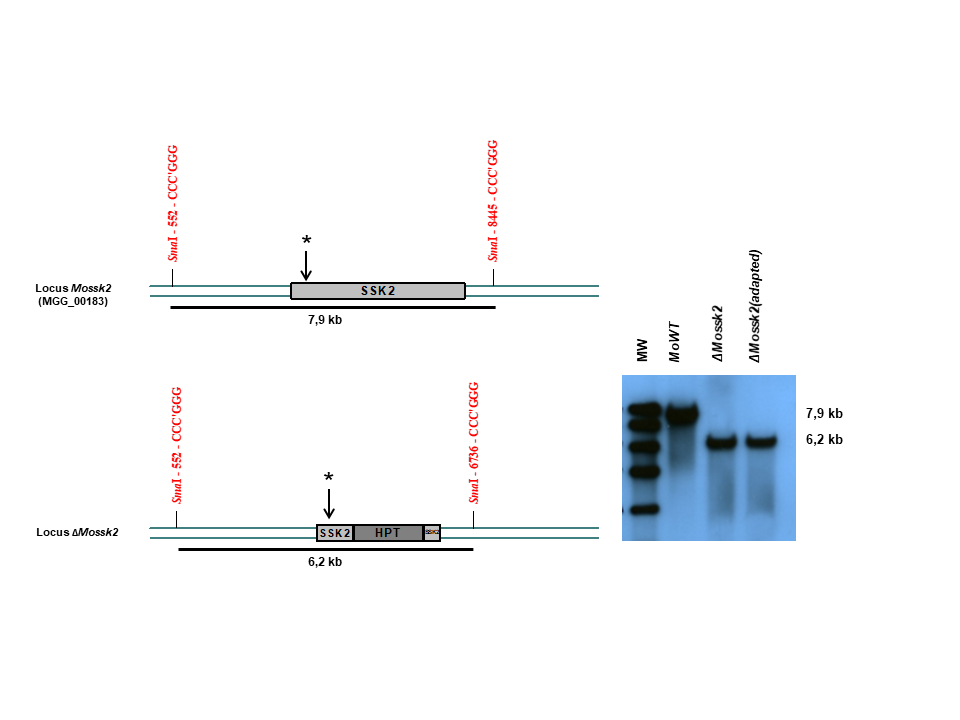


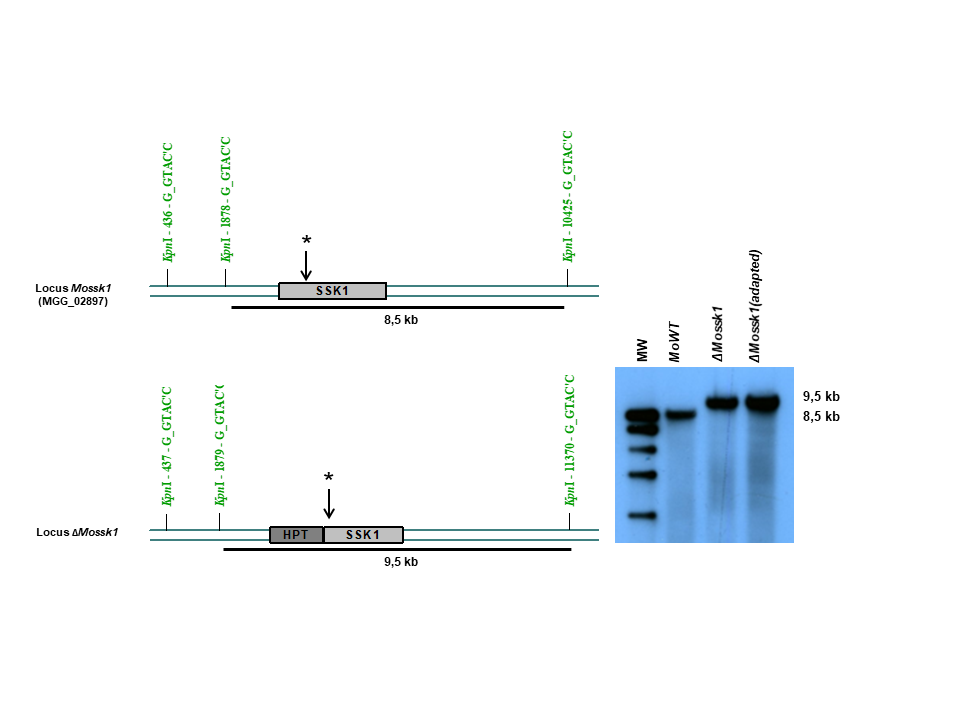


**Figure S1 (continued): Schematic presentation and verification of the *MoWT*, the lof-mutants and the adapted strains by southern hybridization within the *Magnaporthe oryzae* genome.**


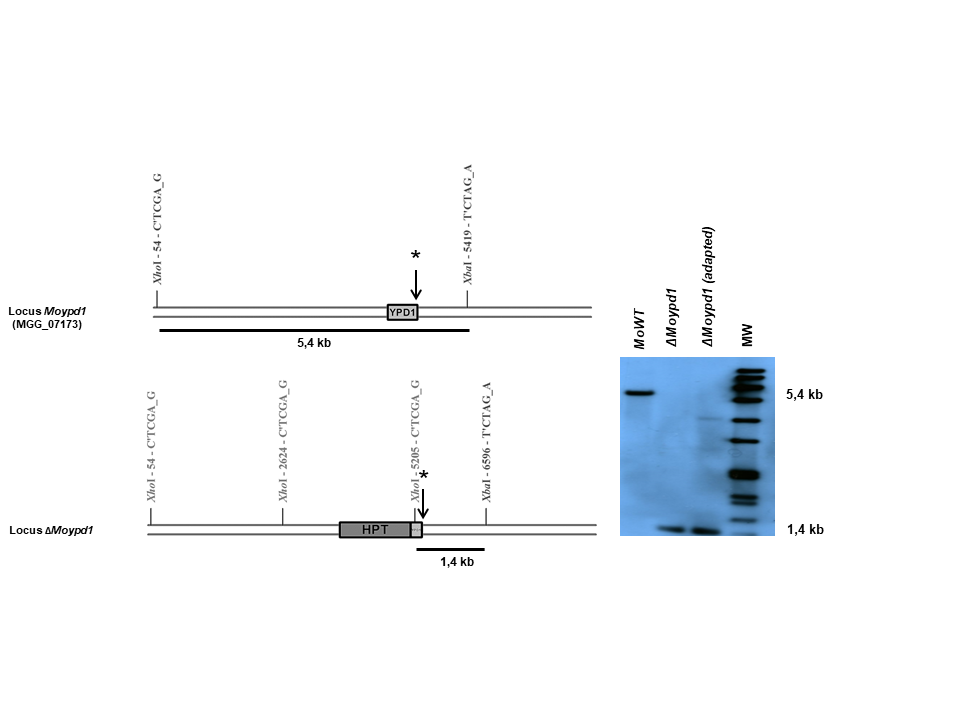


**Figure S1 (continued): Schematic presentation and verification of the *MoWT*, the lof-mutants and the adapted strains by southern hybridization within the *Magnaporthe oryzae* genome.**
