# Supplementary material for: Rapid adaptation of signaling networks in the fungal pathogen Magnaporthe oryzae
Source: BMC Genomics. 2019 Oct 22;20:763. doi: 10.1186/s12864-019-6113-3 (PMC6805500; doi:10.1186/s12864-019-6113-3)
Supplement: Supplementary file 3 — Additional file 3: Figure S2. Investigation of the “adaptation-frequency” in Magnaporthe oryzae mutants with inactivated components of the HOG signaling cascade. [file 12864_2019_6113_MOESM3_ESM.docx]

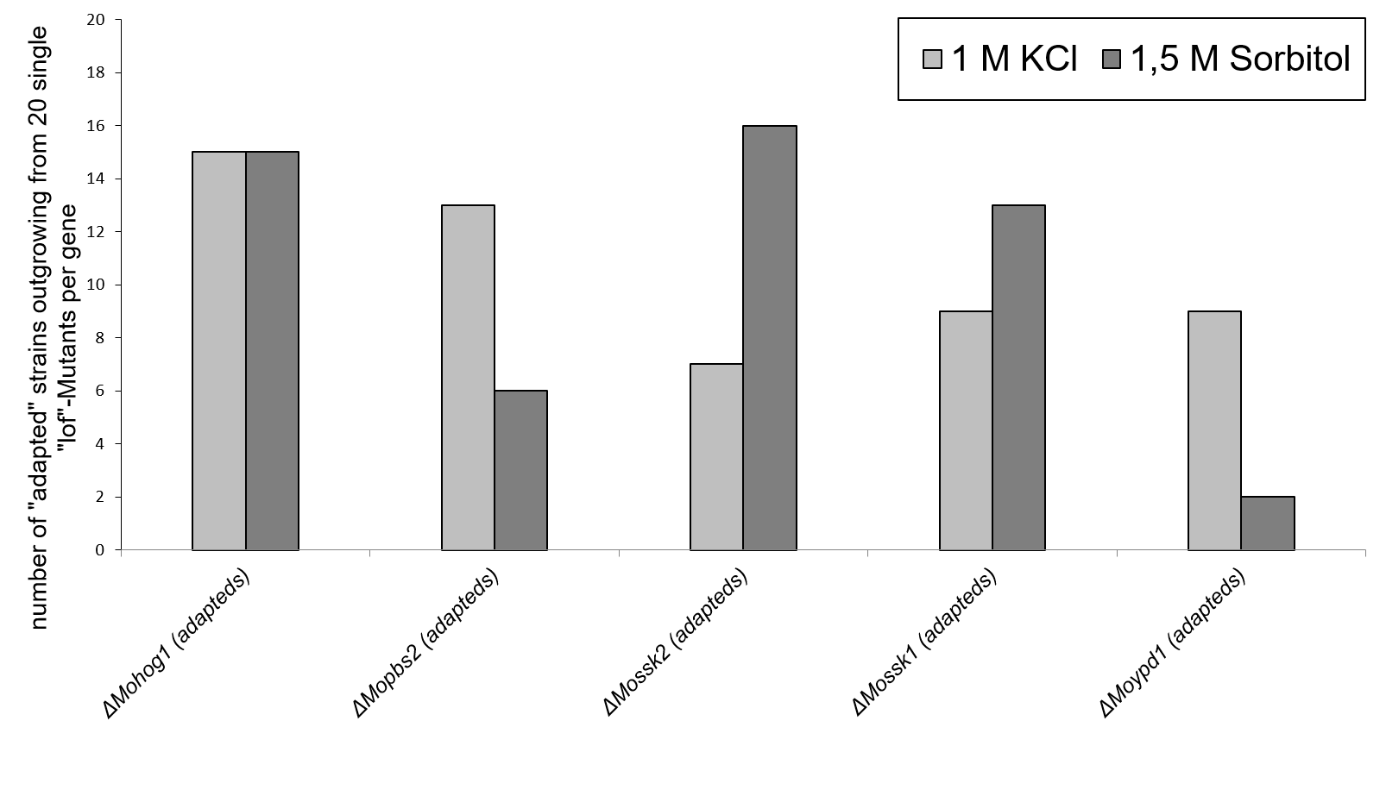


**Figure S2: Investigation of the “adaptation-frequency” in *Magnaporthe oryzae* mutants with inactivated components of the HOG signaling cascade.**
