# Supplementary material for: Rapid adaptation of signaling networks in the fungal pathogen Magnaporthe oryzae
Source: BMC Genomics. 2019 Oct 22;20:763. doi: 10.1186/s12864-019-6113-3 (PMC6805500; doi:10.1186/s12864-019-6113-3)
Supplement: Supplementary file 4 — Additional file 4: Figure S3. Mycelium dry weight of the Magnaporthe oryzae wildtype strain, mutants with inactivated components of the HOG signaling cascade and the “adapted” strains after growth in liquid culture upon sorbitol-stress. The fungal colonies were grown in 250 ml complete medium inclusive 1,5 M sorbitol for 6 d at 26 °C and 120 rpm. Error bars represent the standard deviation of three biological replicates of each strain. [file 12864_2019_6113_MOESM4_ESM.docx]

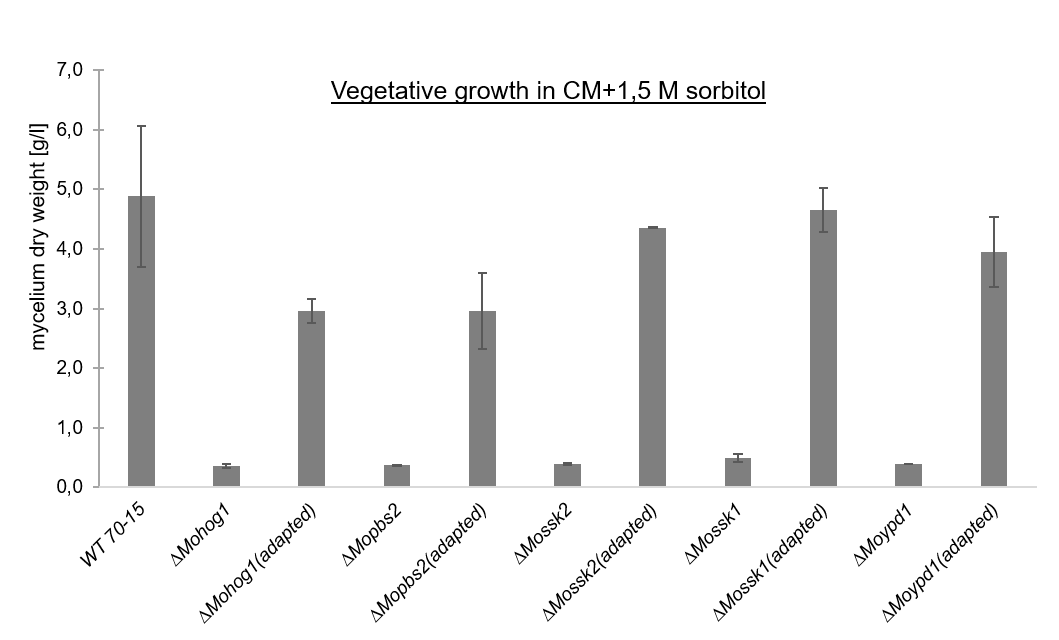


**Figure S3: Mycelium dry weight of the *Magnaporthe oryzae* wildtype strain, mutants with inactivated components of the HOG signaling cascade and the “adapted” strains after growth in liquid culture upon sorbitol-stress.** The fungal colonies were grown in 250 ml complete medium inclusive 1,5 M sorbitol for 6 d at 26 °C and 120 rpm. Error bars represent the standard deviation of three biological replicates of each strain.
