# Supplementary material for: Rapid adaptation of signaling networks in the fungal pathogen Magnaporthe oryzae
Source: BMC Genomics. 2019 Oct 22;20:763. doi: 10.1186/s12864-019-6113-3 (PMC6805500; doi:10.1186/s12864-019-6113-3)
Supplement: Supplementary file 5 — Additional file 5: Figure S4. Pathogenicity assay of the MoWT, the lof mutants and the “adapted” strains. The plant infection assays were carried out as described in experimental procedures. The error bars represent the standard deviation of three experiments with three replicates each. [file 12864_2019_6113_MOESM5_ESM.docx]

**
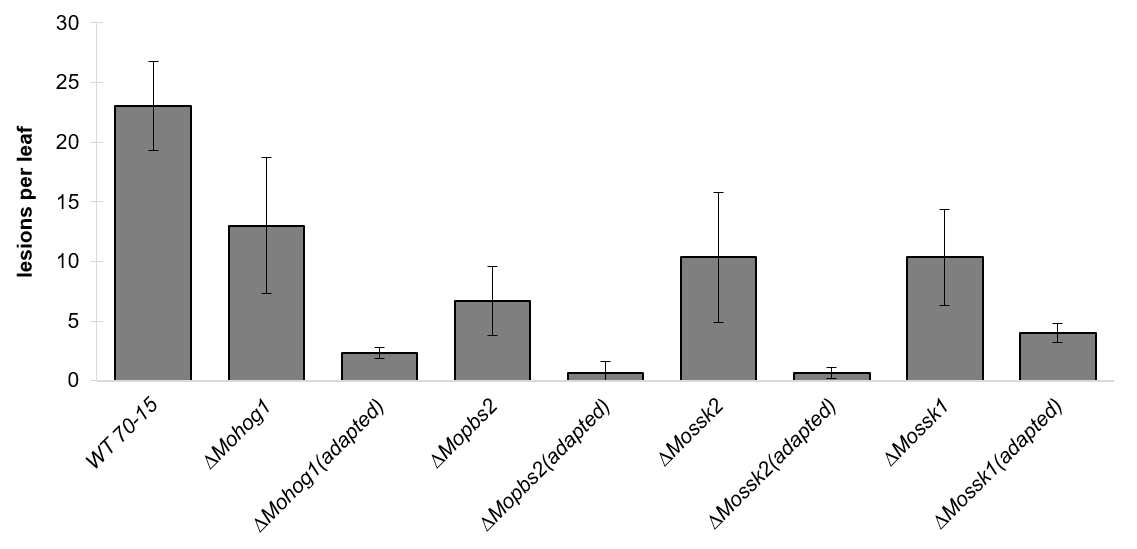
**

**Fig.S4:** **Pathogenicity assay of the *MoWT*, the lof mutants and the “adapted” strains.** The plant infection assays were carried out as described in experimental procedures. The error bars represent the standard deviation of three experiments with three replicates each.
