# Supplementary material for: Rapid adaptation of signaling networks in the fungal pathogen Magnaporthe oryzae
Source: BMC Genomics. 2019 Oct 22;20:763. doi: 10.1186/s12864-019-6113-3 (PMC6805500; doi:10.1186/s12864-019-6113-3)
Supplement: Supplementary file 6 — Additional file 6: Figure S5. VENN diagram of putative structural variations in promotor [A] and in coding sequences (CDS) [B] within the genome of ΔMohog1, ΔMohog1(adapted) and ΔMopbs2(adapted). Numbers in the intersection regions represent overlapping SNPs among the strains. Numbers in parentheses show the corresponding relative percentage of genes harbouring the SNPs. [file 12864_2019_6113_MOESM6_ESM.docx]

**
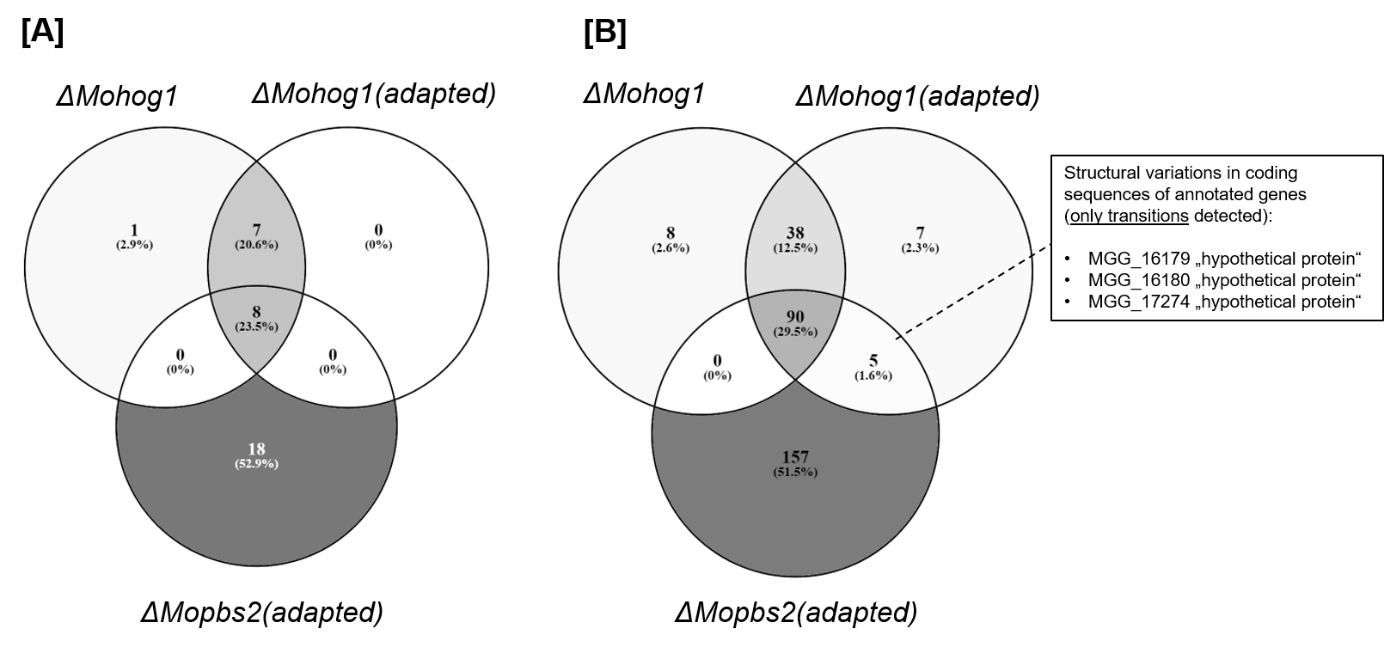
**

**Fig.S5:** **VENN diagram of putative structural variations in promotor [A] and in coding sequences (CDS) [B] within the genome of *ΔMohog1,* *ΔMohog1(adapted)* and *ΔMopbs2(adapted)*.** Numbers in the intersection regions represent overlapping SNPs among the strains. Numbers in parentheses show the corresponding relative percentage of genes harbouring the SNPs.
