# Supplementary material for: Rapid adaptation of signaling networks in the fungal pathogen Magnaporthe oryzae
Source: BMC Genomics. 2019 Oct 22;20:763. doi: 10.1186/s12864-019-6113-3 (PMC6805500; doi:10.1186/s12864-019-6113-3)
Supplement: Supplementary file 7 — Additional file 7: Figure S6. qPCR results of selected genes. qRT-PCR analysis of putative genes in MoWT, the “lof” mutants ΔMohog1 and ΔMohog1(adapted). The M. oryzae cultures were grown for 96 h in CM at 26 °C and 100 rpm. Each of the cultures was separated into two samples, one mixed with 0.5 M KCl and one untreated control further grown in CM at 26 °C and 100 rpm). Samples were taken after 25 min. The RNA was isolated from the mycelium samples and the results of transcript abundance given relative to quantification in the MoWT untreated control. Three biological replicates were used of each. [file 12864_2019_6113_MOESM7_ESM.docx]

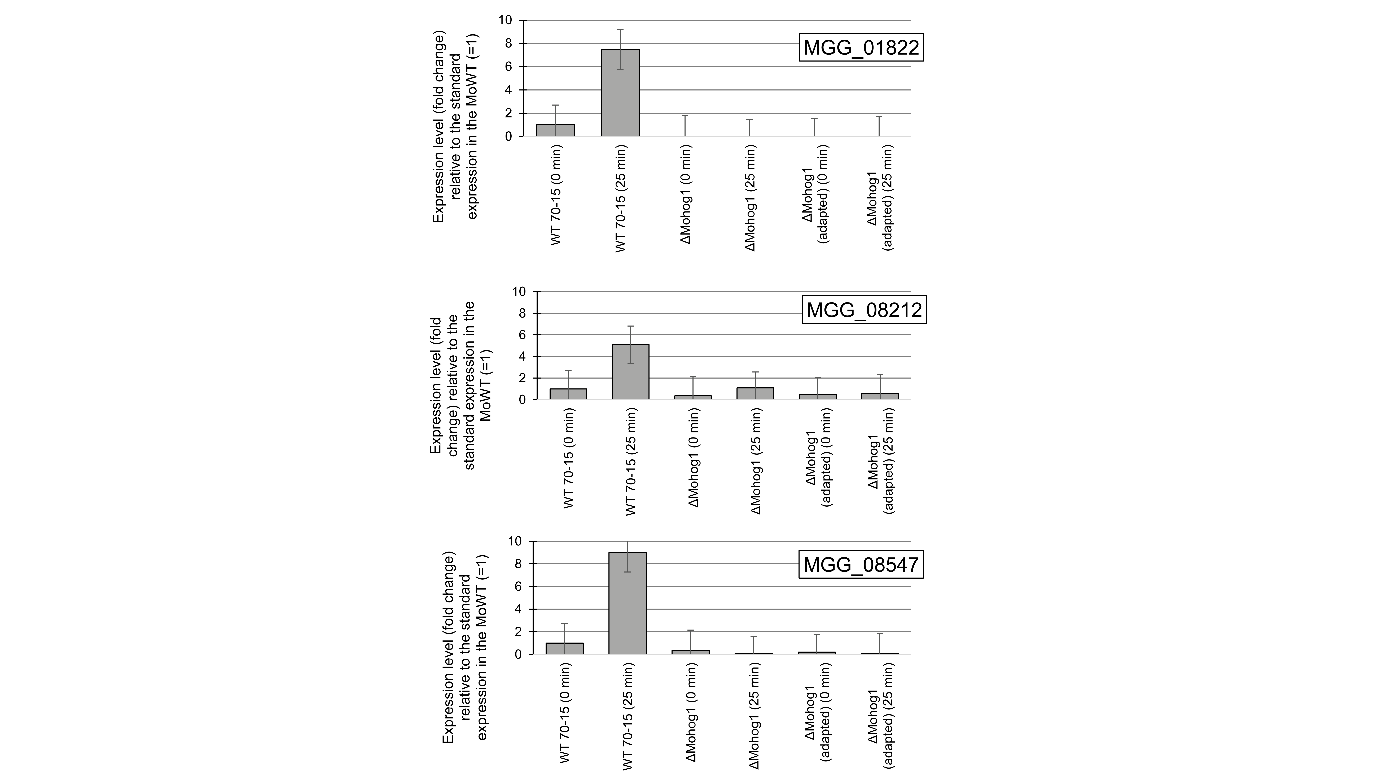


**Fig.S6:** **qPCR results of selected genes**. qRT-PCR analysis of putative genes in MoWT, the “lof” mutants ΔMohog1 and ΔMohog1(adapted). The M. oryzae cultures were grown for 96 h in CM at 26 °C and 100 rpm. Each of the cultures was separated into two samples, one mixed with 0.5 M KCl and one untreated control further grown in CM at 26 °C and 100 rpm). Samples were taken after 25 min. The RNA was isolated from the mycelium samples and the results of transcript abundance given relative to quantification in the MoWT untreated control. Three biological replicates were used of each.
